# Selection of viral variants with enhanced transmission and reduced neutralization susceptibility alongside lateral introductions may explain the persistence of porcine reproductive and respiratory syndrome virus in vaccinated breeding herds

**DOI:** 10.1093/ve/veae041

**Published:** 2024-05-17

**Authors:** Hepzibar Clilverd, Yanli Li, Gerard Martín-Valls, Laia Aguirre, Marga Martín, Martí Cortey, Enric Mateu

**Affiliations:** Department of Animal Health and Anatomy, Universitat Autònoma de Barcelona, Cerdanyola del Vallès 08193, Spain; Department of Animal Health and Anatomy, Universitat Autònoma de Barcelona, Cerdanyola del Vallès 08193, Spain; Department of Animal Health and Anatomy, Universitat Autònoma de Barcelona, Cerdanyola del Vallès 08193, Spain; Department of Animal Health and Anatomy, Universitat Autònoma de Barcelona, Cerdanyola del Vallès 08193, Spain; Department of Animal Health and Anatomy, Universitat Autònoma de Barcelona, Cerdanyola del Vallès 08193, Spain; Department of Animal Health and Anatomy, Universitat Autònoma de Barcelona, Cerdanyola del Vallès 08193, Spain; Department of Animal Health and Anatomy, Universitat Autònoma de Barcelona, Cerdanyola del Vallès 08193, Spain

**Keywords:** PRRSV, endemic, viral evolution, persistence, vaccination

## Abstract

This study investigates the long-term evolutionary dynamics of porcine reproductive and respiratory syndrome virus (PRRSV-1) in an endemically infected and vaccinated pig herd. Over a one year and a half period, piglets from seven farrowing batches in a 300-sow PRRSV-vaccinated farm were monitored from birth to nine weeks of age by reverse transcription-quantitative polymerase chain reaction (RT-qPCR). Eighty-five PRRSV-positive samples were subjected to whole genome sequencing (Illumina Miseq), and 251 samples to open reading frame 5 (ORF5) sequencing. Farm-specific PRRSV variants’ impact on anti-PRRSV antibodies was evaluated using enzyme-linked immunosorbent and neutralizing antibody assays. The replication kinetics and cytokine inhibition capabilities (IFN-α and TNF-α) of these variants were assessed in porcine alveolar macrophages. The study revealed fluctuating PRRSV-1 incidences in farrowing units and nurseries, attributed to two key evolutionary events: an escape variant emergence and a lateral introduction of a new strain. Initially, strain 1 variant α was swiftly replaced within weeks by variant 1β (99.5 per cent genomic similarity), with twenty-five amino acid mutations, primarily in nsp1α, GP2, GP3, and GP5, including an additional glycosylation site and a deletion downstream the neutralization epitope of GP5. This shift to 1β correlated with increased incidence in nurseries and higher viral loads, with sera from 1α-exposed animals showing reduced neutralization against 1β. Consistently for *in vitro* assays, variant 1β demonstrated enhanced replication in porcine alveolar macrophages but no difference regarding IFN-α or TNF-α responses. Later, a new strain (strain 2, 83.3 per cent similarity to strain 1) emerged and led to incidence resurgence because of the low cross reactivity with the previous antibodies. The study highlights PRRSV’s rapid adaptability and challenges in controlling its spread, underscoring the necessity for more effective vaccines and eradication approaches.

## 1. Introduction

Maintaining high health standards in modern pig production is essential for economic sustainability and ethical considerations. Porcine reproductive and respiratory syndrome (PRRS), one of the costliest and welfare-impacting diseases in pigs (Neumann et al. 2005; Holtkamp et al. 2013; Nathues et al. 2017), exemplifies these challenges.

Caused by two species, *Betaarterivirus suid 1* and *Betaarterivirus suid 2*, commonly known as PRRS virus 1 (PRRSV-1) and PRRSV-2, respectively, PRRSV is characterized by its high genetic diversity. This diversity arises from a combination of factors: high-rate substitution, frequent recombination, worldwide spread, and immune selection pressures (Forsberg 2005; Martín-Valls et al. 2014). Such genetic and antigenic diversity facilitates infection in herds with pre-existing immunity from either prior infections, vaccinations, or both. Noteworthy, neutralizing antibodies targeting PRRSV seem to be strain-specific and have limited cross-reactivity (Martínez-Lobo et al. 2011).

Upon initial viral introduction in a naïve sow farm, a reproductive outbreak typically ensues, characterized by late-gestation abortions, premature or delayed farrowing, a rise in mummified foetuses and stillbirths, and an increase in weak-born piglets along with higher mortality rates in the farrowing units (Zimmerman et al. 2019). In cases of highly virulent strains, sow mortality is also observed (Halbur and Bush 1997; Tian et al. 2007; Martín-Valls et al. 2023). The infection then progresses to nurseries, manifesting as respiratory disease, often exacerbated by secondary pathogens (Zimmerman et al. 2019).

A pivotal aspect of PRRS epidemiology is the vertical transmission during late gestation, resulting in the birth of viraemic piglets that can remain infectious for months and act as primary infection sources for other litters, in both the farrowing units and nurseries (Benfield et al. 2000; Rowland et al. 2003). Thus, vertical transmission is thought to be the primary contributor to the maintenance of infection within a farm (Pileri and Mateu 2016). Consequently, most monitoring programmes focus on detecting viral circulation in the farrowing units, using terms ‘unstable’ or ‘stable’ to indicate the virus’s presence or absence, respectively (Holtkamp et al. 2011, 2021). Monitoring is typically achieved by examining pigs at weaning or testing individual or aggregate samples, such as umbilical cords (UCs), tongue tips, and processing fluids, from piglets in the farrowing units (Martín-Valls et al. 2018; Vilalta et al. 2018; Baliellas et al. 2021).

In the absence of intervention, PRRS often becomes endemic, with periodic reproductive and respiratory disease rebounds (Pileri and Mateu 2016). Given the widespread nature of the virus, novel introductions of different PRRSV strains, referred to as ‘lateral introductions’, are common. The consequences of such introductions vary widely, ranging from subclinical infections to severe outbreaks, and are influenced by the strain’s virulence and the existing immunity’s cross-reactivity.

Control of PRRSV requires a multifaceted approach that encompasses vaccination, biosecurity, animal movement management, as well as accurate monitoring and diagnostics. Vaccination of sows is a common practice, starting with gilts and followed by recall vaccinations every few months. Live attenuated vaccines are typically employed due to limited efficacy of the inactivated ones (Zuckermann et al. 2007).

However, even with robust vaccination and biosecurity protocols, PRRSV can persist within herds. Unfortunately, there is limited understanding of the specific mechanisms allowing the virus to persist within these populations, hindering the development of alternative strategies beyond resorting to herd depopulation or closure, both of which carry substantial economic implications (Torremorell, Henry, and Christianson 2003; Corzo et al. 2010).

This study aims to elucidate long-term evolutionary patterns of PRRSV in farms where the virus persists with conventional control measures. We closely monitored an endemically infected farm for over one year and a half, tracking piglets from birth to the end of their nursery period. Our focus was on unravelling the temporal evolution of the virus and correlating it with the epidemiological impacts, humoral responses, and potential mechanisms of immune evasion. Insights from this study would be crucial for developing more effective control strategies in farms grappling with endemic PRRSV infections.

## 2. Materials and methods

### 2.1 Study farm and follow-up chronology

The study farm housed a total of 300 breeding sows, which were managed using a three-week batch farrowing system. Piglets were weaned at four weeks of age and then raised in nurseries until nine weeks of age before transferring to a fattening unit to reach market weight (100 kg). Before the study commenced, the farm had a history of routine PRRSV positivity (for years) in weaned pigs as identified by reverse transcription-quantitative polymerase chain reaction (RT-qPCR).

The breeding herd and nurseries were in the same location, while the fattening units were in a different location and managed by another farmer. The farm made some biosecurity improvements during the study. Clear delimitation between clean and dirty areas for visitors was established, and a complete perimeter fence was constructed.

All piglets received colostrum exclusively from their mother, except in cases of birthing complications such as uterine prolapse or sudden death. In such rare instances (only one reported during the study), piglets were allocated to sows that had given birth within the preceding 24 h, when available.

Replacement gilts, purchased from a PRRSV-negative herd at six months of age, were allocated at their arrival to a separate building within the farm’s perimeter. They underwent a quarantine period of no less than six weeks until their first service. Gilts received at least two doses of a modified live PRRSV vaccine (Porcilis® PRRS, MSD, Spain) prior to their first service, with a minimum two-week interval between the second dose and exposure to other sows in the breeding herd. A blanket vaccination protocol was implemented for all sows and gilts, involving at least three vaccinations annually. Vaccination was not routinely confirmed by serology in all individuals.

**Figure 1.**
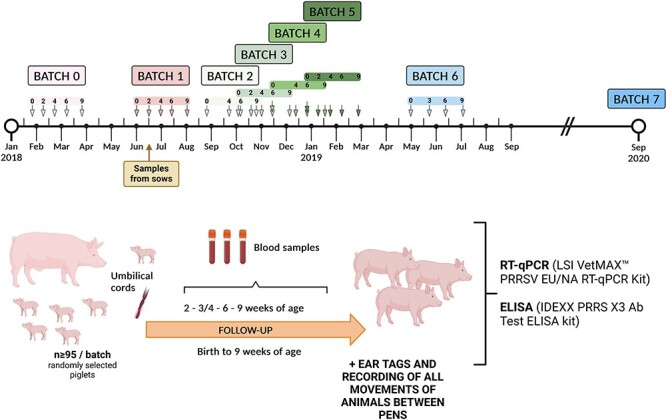
Graphical depiction of the sampling scheme in the farm. Seven batches of piglets (numbered 0 to 6) were followed periodically from birth to nine weeks of age to assess the dynamics of PRRSV infection in the farm. At least ninety-five piglets were randomly selected in each batch. UCs were collected at birth followed by periodic blood sampling. Samples were analysed by RT-qPCR and ELISA to assess the infection status. An additional sampling (Batch 7) was conducted fourteen months after Batch 6. Serum samples from fourteen sows in Batch 1 were collected two weeks after farrowing. Created with BioRender.com.

Figure 1 depicts the sampling scheme employed in this study. The initial farm sampling (Batch 0) was followed by six subsequent batches (Batches 1–6, from May 2018 to July 2019) to monitor PRRSV status. Each sampling involved monitoring at least ninety-five out of an average of 400 piglets in total in each batch (about forty-one farrowings per batch), from sows of various parities, from birth to nine weeks of age. This sample size allowed to detect a ≥3 per cent infection rate with 95 per cent confidence. UCs were collected at birth, and blood samples were obtained at various time points: once or twice in the farrowing unit (at three or four weeks of age for Batches 2, 4, and 6, and at two and four weeks of age for Batches 1, 3, and 5) and twice in the nurseries (at six and nine weeks of age). Each animal was ear-tagged for identification. An additional sampling (Batch 7, September 2020) was conducted fourteen months after Batch 6 sampling (thirty-two months after Batch 0 sampling), with UCs collected at birth and blood samples collected from three-, six-, and nine-week-old animals. The farm’s productivity data and mortality rates were recorded throughout the study period.

In addition, serum samples from fourteen sows in Batch 1 were collected two weeks after farrowing. Moreover, tonsils from ∼10 per cent of sows (twenty-eight animals) were collected from animals sent to the slaughterhouse at different time points during the study period.

The animal study was reviewed and approved by the Ethics Committee in Animal and Human Research of the Universitat Autònoma de Barcelona (Approval Numbers 3221‐CEEA‐UAB and CEEAH‐5691 for the project and the procedure, respectively).

### 2.2 PRRSV detection

Upon arrival at the laboratory, the UC and blood samples were processed as previously described (Martín-Valls et al. 2018; Clilverd et al. 2023). Briefly, UCs were minced into 5 ml of minimal essential medium, centrifuged at 12,000 × g for 5 min, and the supernatant was aliquoted and stored at −80 °C. Blood samples were centrifuged at 300 × g for 5 min and subsequent serum aliquots were preserved at −80 °C.

Viral RNA extraction was performed using NucleoSpin® RNA virus kit (Macherey-Nagel, Germany) with a final elution volume of 50 µl. Each extraction included a PRRSV positive sample and DEPC-treated water as controls. PRRSV RNA detection was performed with a commercial RT-qPCR kit designed to detect both PRRSV-1 and PRRSV-2 (LSI VetMAX™ PRRSV EU/NA kit, Thermo Fisher Scientific) with an internal positive control included in each sample. In order to increase the specificity of the test, samples yielding *C*_t_ values ≤37 were considered positive, while those with *C*_t_ values between 37.1 and 39.9 were deemed suspicious. Suspicious samples, upon retesting, were considered negative if *C*_t_ values remained ≥37.1.

Tonsil homogenates were subject to RNA extraction with TRIzol reagent and RT-qPCR analysis as described earlier.

Vertical transmission events (VTEs) were identified based on the detection of at least one positive UC per litter using RT-qPCR. Incidence rates within the monitored group of animals (*n* ≥ 95) were calculated by dividing new cases by the number of susceptible animals during each observation period. If an animal tested positive in two consecutive samplings, it was categorized as a new case only at the first positive sampling time. Moreover, to account for infected animals that could have been undetected, pigs seropositive at nine weeks of age without prior RT-qPCR detection were also considered new cases. Animals with missing data were excluded from the calculations.

### 2.3 PRRSV isolation

Porcine alveolar macrophages (PAMs), sourced from high-health-status pigs via bronchoalveolar lavage (Mayer and Lam 1984), were used for PRRSV isolation from RT-qPCR-positive samples. Prior to usage, PAMs were tested negative for PRRSV, porcine circovirus 2, and mycoplasma by RT-qPCR. In each followed pig batch, viral isolation was attempted only from samples (all UC and at least 20 per cent of the blood samples) with *C*_t_ values ≤32.0. The isolation was restricted to a single passage to prevent any potential bias in the results of the whole genome sequencing (Cortey et al. 2018). On observing the cytopathic effect, cell culture supernatants were collected, centrifuged at 400 × g for 10 min, aliquoted, and preserved at −80 °C until use.

### 2.4 Sequencing

To gain a comprehensive understanding of the viral genetic variability and evolutionary patterns, at least 20 per cent of the positive samples from every batch were randomly selected for whole genome sequence analysis. After viral isolation of these samples, viral RNA was extracted from the collected cell culture supernatants using the TRIzol™ reagent with a 20-µl elution volume. Isolates were then sequenced using Illumina Miseq technology, following a previously described protocol (Clilverd et al. 2023) with no prior amplification steps involved. A total of eighty-five PRRSV isolates were subjected to next generation sequencing.

All RT-qPCR-positive samples with a *C*_t_ value ≤32.0 underwent amplification and Sanger sequencing of open reading frame 5 (ORF5) (*n* = 251 out of the 505 positive samples with a *C*_t_ value ≤37.0), which encodes for the virus major envelope protein and has conventionally been employed for phylogenetic analysis of PRRSV. A previously described protocol (Mateu, Martín, and Vidal 2003) and tailor-made oligonucleotide reverse primers were used (Supplementary Table S1).

The consensus sequences obtained from both whole genome and the ORF5 sequencing were submitted to GenBank with the Accession Numbers OR667160 to OR667244 and OR620633 to OR620890, respectively.

### 2.5 Phylogenetic and evolutionary analysis

To elucidate the genetic relationships and diversity of the obtained PRRSV sequences in this study, a phylogenetic analysis was conducted that encompassed the whole genome and ORF5 sequences. For phylogenetic tree construction, Bayesian inferences through MrBayes (available at https://ngphylogeny.fr) with one million iterations were employed. To quantify genetic variations, *p*-distances were calculated using MEGA X (Kumar et al. 2018), enabling the assessment of inter- and intra-batch and viral variant diversity for both the whole genome and ORF5 sequences. In addition, the potential occurrence of recombination events in the whole genome sequences was investigated employing the GARD algorithm method (Pond et al. 2006) and RDP5.0 software (Martin et al. 2021). Furthermore, a comparative analysis of the predicted protein sequences’ amino acid composition was conducted from the consensus sequences, enabling a detailed examination of differences and similarities between the viral strains and variants. Moreover, to analyse the presence of subsequent mutations in 1α, the nucleotide frequency per position was determined for all sequences from Batches 0 and 1, and the proportion of different amino acids within the quasi-species was inferred.

A collection of complete genomes and ORF5 sequences of PRRSV-1 strains, which included the five PRRSV vaccines that have been commercially licensed in Spain, were retrieved from GenBank (Supplementary Table S2). To conduct a comparative analysis, phylogenetic trees were constructed using MrBayes and genetic distances were quantified (*p*-distance) as mentioned earlier.

Prediction of *N*-glycosylations in the viral structural glycoproteins was carried out using Net-N-Glyc (Gupta and Brunak 2002) available at https://services.healthtech.dtu.dk/services/NetNGlyc-1.0/.

### 2.6 Adaptation to grow on MARC-145 cells and viral production in PAM

Viruses successfully isolated from serum samples were subjected to adaption in MARC-145 cells. Confluent monolayers of MARC-145 cells in 24-well plates were inoculated with RT-qPCR positive isolates. The isolate for each viral variant (1α, 1β, and 2) that adapted to MARC-145 and yielded the higher titre was selected for further experiments, with the fifth passage designated as the working stock. Titration of the viral stocks was performed by the TCID_50_ method, following the Reed and Muench (1938) calculation. To identify mutations associated with the virus’s adaptation to MARC-145 cells, whole genome sequences were obtained from the MARC-145-adapted viruses (Passage 5), as described in Section 2.4, and, subsequently, a sequence comparison with the original sample was performed. The consensus sequences of variants 1α, 1β, and 2 were submitted to GenBank, corresponding to the Accession Numbers OQ440239, OQ440240, and OQ440241, respectively. Supplementary Table S3 shows the differences between the PAM-isolated and MARC-145-adapted isolates.

The original isolates for which MARC-145 adaptation was successful were then used for propagation in PAM to generate a high-titre virus stock for subsequent experiments. The fifth passage of 1α, 1β, and 2 in PAM served as the working stock for further experiments.

### 2.7 Serological analyses (enzyme-linked immunosorbent assay and viral neutralization test)

All serum samples collected in the farrowing units and from nine-week-old animals were analysed by enzyme-linked immunosorbent assay (ELISA) to determine the presence of anti-PRRSV antibodies (IDEXX PRRS X3 Ab Test, IDEXX, USA). Furthermore, over 60 per cent of six-week-old animals in each batch were randomly selected for the same analysis. The ELISA results were expressed as sample to positive control (*S*/*P*) optical density ratios according to the manufacturer’s instructions. *S*/*P* ratios ≥0.40 were considered positive.

The presence of neutralizing antibodies was assessed against the three viral variants identified in this study (1α, 1β, and 2) and the vaccine strain employed on the farm. For this purpose, a subset of serum samples from two-week-old animals in Batches 1, 5, 6, and 7 was chosen to examine maternally derived antibody titres. These particular batches were selected as only one viral variant was circulating in their respective farrowing units at that time. Titres of neutralizing antibodies of those samples were assessed against the circulating viral variant in the corresponding batch. For Batches 1 and 5, sera were also tested against the subsequently appearing variant or strain. In addition, serum samples from fourteen sows in Batch 1 were tested against the circulating viral variant in that batch and the emerging one in Batch 2. All samples were further tested against the vaccine virus used in the farm, serving as a reference for comparison.

To determine antibodies in piglets after the infection by a given viral variant, nine-week-old animals that had been infected before six weeks of age were selected, ensuring sufficient time for the development of neutralizing antibodies. In this case, the assessment of neutralizing antibodies was performed against the three viral variants but not the vaccine, as piglets were not vaccinated.

The viral neutralization test (VNT) was conducted using a previously described protocol (Yoon et al. 1994) with minor modifications. Briefly, VNT was performed in MARC-145 cell monolayers. The neutralization titre was initially assessed by determining the reciprocal of the highest serum dilution that resulted in complete inhibition of the cytopathic effect. Samples were tested in duplicate, and the titre of a sample was calculated as the average of two replicas. Samples with replicas that differed more than one dilution were retested. Neutralizing antibody titres were expressed as log_2_ values with titres ≥3 log_2_ considered positive. To confirm neutralization, cell cultures were fixed in 150 µl of methanol–ethanol (75:25) at −20 °C for a minimum of 15 min. Then, cells were stained with anti-PRRSV-1 nucleocapsid protein (N) antibody (clone 1C5H; Ingenasa, Madrid, Spain) followed by a secondary fluorescein-labelled goat anti-mouse IgG2b (H + L) (Jackson ImmunoResearch, Spain), and checked using an inverted fluorescence microscope (Optika® Italy IM-3FL, Optika S.r.l, Italy).

### 2.8 Viral replication kinetics

The replication kinetics of viral variants 1α and 1β were evaluated in PAM. Briefly, PAMs were seeded overnight in 96-well plates at a density of 1.2 × 10^5^ cells/well. Then, they were inoculated with each variant at a multiplicity of infection (MOI) of 0.1. After 90 min incubation, unbound virus was washed away and the cultures were replenished with fresh culture medium, being incubated for different times (0, 12, 24, 48, and 72 h). Supernatants were collected to assess the extracellular virus, while cells were fixed in 150 µl of methanol–ethanol (75:25) at −20 °C for further analysis.

RNA from the supernatants was extracted using MagMax Core Nucleic Acid Purification Kit (Applied Biosystems, Thermo Fisher Scientific, USA), following the manufacturer’s instructions. The quantification of viral loads was performed using a commercial RT-qPCR kit, as described in Section 2.2. A standard curve correlating *C*_t_ values to viral loads was established using a decimal dilution series of each viral isolate, tested in triplicates.

Furthermore, the proportion of infected PAM at each time point was determined using the fixed PAM cultures. For this purpose, cells were labelled with mAb 1CH5 along with a secondary fluorescein-labelled goat anti-mouse IgG2b as for the neutralization test (Section 2.7). Nuclear staining was performed with Prolong^TM^ Gold Antifade Mountant with DAPI stain (Invitrogen, Thermofisher Scientific, USA). PRRSV-positive cells were counted in five fields per replicate (400× magnification) with at least 100 cells per field, using an inverted fluorescence microscope (Optika® Italy IM-3FL, Optika S.r.l, Italy).

### 2.9 Attachment of different viral variants to PAM

To explore factors influencing the replication kinetics of viral variants, the assessment of the attachment to PAM was conducted. PAMs were seeded overnight, then detached and transferred to 1.5 ml tubes (65,000 cells/tube), where cells were inoculated with each variant at an MOI of 1 on ice for 60 min. Unbound virus was washed away, and cells were fixed with cold methanol–ethanol (75:25) at −20 °C for a minimum of 20 min. Three replicates were prepared for each variant, and mock-inoculated cells were used as the negative control.

Immunofluorescence staining along with NucBlue nuclear staining was performed. PRRSV-1 N was labelled with primary antibody 1CH5 (Ingenasa, Spain), followed by a secondary antibody anti-mouse IgG2b conjugated to Alexa Fluor 647 (Invitrogen, Thermo Fisher Scientific, USA). Negative controls included time zero samples, mock-inoculated cultures, and irrelevant mouse IgG2b isotype-matched antibody staining (Bio-Rad, UK). After staining, slides were mounted with Prolong Glass Antifade Mountant with NucBlue (Invitrogen, Thermo Fisher Scientific, USA). Confocal analysis was performed using a Leica TCS SP5 confocal microscopy. Channel merging and image processing were conducted using Fiji software (Schindelin et al. 2012).

### 2.10 Inhibition of IFN-α and TNF-α

To determine whether differences in viral replication among different viral variants could be attributed to their differential impact on antiviral cytokines, IFN-α and TNF-α were examined. PAMs were overnight seeded in 96-well plates (1 × 10^5^ cells/well) followed by stimulation with different viral variants at MOI 0.5, 10 µg/ml poly I:C (InvivoGen, USA), or culture medium, in various combinations: (1) virus only, (2) poly I:C only, (3) virus and poly I:C simultaneously, (4) virus followed by poly I:C 6 h after the inoculation of the virus, (5) poly I:C followed by the virus 6 h later, and (6) culture medium. After 24 h of stimulation, cell culture supernatants were collected and examined by ELISA to measure IFN-α and TNF-α levels (Porcine IFN-α ELISA kit and TNF α Porcine ELISA kit, Invitrogen, Thermo Fischer Scientific, USA). The optical densities from mock-inoculated cultures were used to evaluate the background of the assay and were subtracted from the obtained values with each stimulus.

### 2.11 Statistical analyses

Statistical analyses were performed using GraphPad Prism v.10. Significance was set to *P* < 0.05. Comparison between incidences of PRRSV infection were performed using the *χ*^2^ (Fisher’s exact test when needed). Comparison of *C*_t_ values, *S*/*P* ratios, or neutralization titres were performed using the Kruskal–Wallis test. Comparison of the proportions of infected cells in the examined replicas were calculated using a Mann–Whitney test. Linear regression analysis was used to correlate incidences between different production phases.

## 3. Results

### 3.1 PRRSV infection dynamics in the farm and VTEs

During the study, a follow-up of 685 piglets from 149 litters was conducted, spanning seven consecutive batches over a period of approximately one and a half years (Batches 0–6). The data regarding the sows and litters followed in each batch have been summarized in Table 1 and Supplementary Table S4. Interestingly, marked variations were observed between the proportions of PRRSV-positive litters at birth, and the incidences recorded in the farrowing units and nurseries (Table 1). It is worth noting that increased proportions of positive litters at birth could not be significantly correlated with increased transmission rates in the farrowing units. Similarly, the incidence in the farrowing units did not correlate with an increased incidence in nurseries, and vice versa. Incidences for each sampling time point, along with additional data, are provided in Supplementary Table S4.

**Table 1.** Basic data on the followed pig batches with indication of the numbers of litters and pigs, parity ranges for the sows, time between batches, incidence of PRRSV infection in farrowing units and nurseries, and the viral variant/strain detected at each batch.

Batch	No. of litters examined at birth/followed	Median (IQR) [range sows’ parities]	Pigs followed (1–9 woa)/number of pigs born alive	Litters with PRRSV+ piglets at birth	Months elapsed since the beginning of the previous batch	Incidence (PCR+) in farrowing units^a^	Incidence (PCR+) in nurseries (+seropositive)^a^	Viral variant/strain present
0	9/9	5 (4–6) [1–8]	72/N.R.^b^	3 (33.3%) (CI_95%_: 9.0–69.1%)	N.A.	1.6% (CI_95%_: 0.1–9.8%)	59.0% (CI_95%_: 47.7–71.2%) (85.3%) (CI_95%_: 36.3–62.2%)	1α
1	32/25	4 (2–6) [1–8]	113/475	6 (18.8%) (CI_95%_: 7.9–37.0%)	4.3	10.3%* (CI_95%_: 4.6–20.7%)	31.2%***(CI_95%_: 20.2–44.4%) (49.2%)****(CI_95%_: 36.3–62.2%)	1α
2	27/24	3 (2–5) [2–9]	98/356	3 (11.1%) (CI_95%_: 2.9–30.3%)	2.8	1.7%* (CI_95%_: 0.1–10.1%)	88.1%*** (CI_95%_: 76.5–94.7%) (93.2%)**** (CI_95%_: 82.7–97.8%)	1β
3	27/18	4 (1–6) [1–7]	97/315	4 (14.8%) (CI_95%_: 4.9–34.6%)	1.3	3.2% (CI_95%_: 0.8–9.6%)	58.7%*** (CI_95%_: 47.9–68.7%) (73.9%)** (CI_95%_: 63.5–82.3%)	1β
4	34/34	4.5 (2–6) [1–7]	115/354	0 (0.0%) * (CI_95%_: 0.0–12.6%)	1.4	1.6%* (CI_95%_: 0.1–9.7%)	16.1%*** (CI_95%_: 8.4–28.1%) (33.9%)**** (CI_95%_: 22.7–47.1%)	1β
5	28/27	1 (1–3) [1–10]	95/355	3 (10.1%) † (CI_95%_: 2.8–29.4%)	1.4	10.5%** (CI_95%_: 5.0–20.2%)	97.1%*** (CI_95%_: 88.8-99.5%) (98.5%)**** (CI_95%_: 91.0–99.9%)	1β/2
6	18/12	1 (1–1) [1–5]	95/361	0 (0.0%) (CI_95%_: 0.0–21.9%)	4.2	1.1%** (CI_95%_: 0.1–6.8%)	65.9%*** (CI_95%_: 55.2–75.3%) (75.8%)**** (CI_95%_: 65.5–83.9%)	2
7^c^	5/0	6 (1–6) [1–7]	N.A.	1 (20%) (CI_95%_: 1.05%–70.12)	16.8	42%^c^ (CI_95%_: 28.5–56.7%)	52.5% ^c^ (CI_95%_: 36.3–68.2%) (62.5%)^c^ (CI_95%_: 50.8–80.9%)	2
**Totals/** **averages**	175/149	3 (1–5) [1–10]	685/> 2,216^b^	10.9%	N.A.	4.3%± 4.2%	59.5%± 28.7% (72.8%± 23.5%)	N.A.

a Differences were calculated with regard to the previous batch.

b The total number of piglets born alive in Batch 0 was not recorded.

c In this case, a cross-sectional sampling was performed. Data represent prevalence not incidence; it has not been included in the total calculated average.

† *P* < 0.10;

* *P* < 0.05;

** *P* < 0.01;

*** *P* < 0.001;

**** *P* < 0.0001.

IQR: interquartile range. N.A.: does not apply. N.R.: not recorded. PCR+: PCR positive. PRRSV+: PRRSV positive. woa: weeks of age

**Figure 2.**
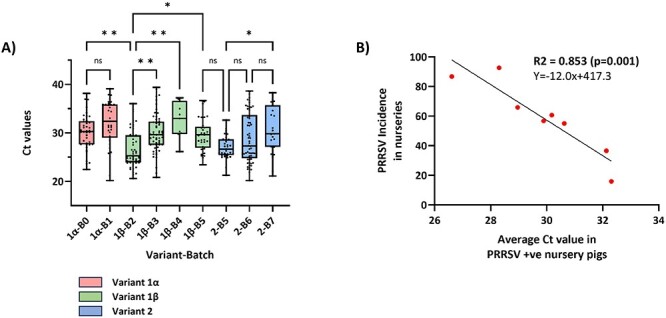
*C*_t_ values of the newly infected piglets in the nurseries (A) and correlation of the *C*_t_ values with the incidence of the infection in each nursery (B). (A) The graph compares *C*_t_ values of RT-qPCR positive newly infected animals in the nurseries of Batches 0–7 (B1–B7). Each dot represents an examined individual. The box and whiskers plot shows the minimum, maximum, median, and quartiles 25 and 75. The *x*-axis shows values for each viral variant/strain. Boxes with the same colour represent the same viral variant/strain. ns = not significant. *P < 0.05; **P < 0.01; ***P < 0.001; ****P < 0.0001. (B) The graph shows the correlation between the average *C*_t_ values of each batch (red dots) with the cumulative incidence in the nursery in that batch.

RT-qPCR allowed to compare the viral loads among animals in each batch. Upon comparing the *C*_t_ values of pigs that were newly infected in the nurseries within each batch, the results showed significant differences (Fig. 2). Specifically, with each rise in nursery incidence, there was a corresponding decrease in the average *C*_t_ values of viraemic animals detected in those nurseries (*R*^2^ = 0.853; *P* = 0.001), thereby implying that increased incidences could be correlated with increased individual viral loads (Fig. 2).

Regarding the examination of sow tonsils for the presence of PRRSV, all but one were negative by RT-qPCR. The positive sow (*C*_t_ = 37) could not be related with a VTE.

### 3.2 Phylogenetic and recombination analyses and viral variant characterization

**Figure 3.**
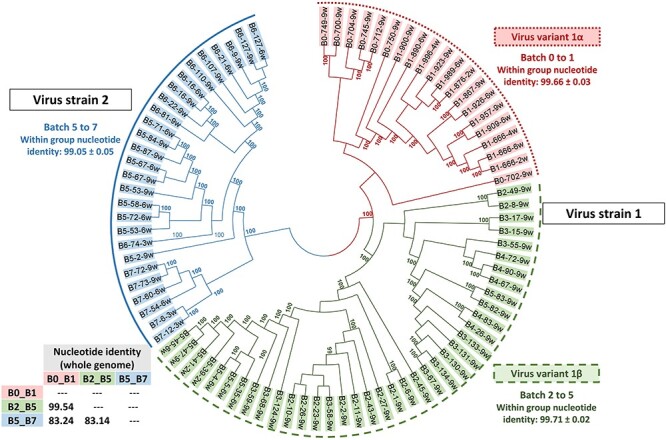
Bayesian tree showing the phylogenetic grouping of the whole genome sequences obtained in this study. Posterior probabilities higher than 70 per cent are shown. All isolates from Batches 0 and 1 belonged to the 1α cluster (dotted line). All isolates of Batches 2–4 and most of the Batch 5 sequences belonged to variant 1β (dashed line). In Batch 5, a new strain appeared in the nurseries and, subsequently in Batches 6 and 7, it became the only detectable PRRSV strain (strain 2, solid line). No statistically significant variants were found for strain 2.

Figure 3 displays a Bayesian phylogenetic tree that was constructed using the whole genome sequences obtained during the study. This analysis initially identified a PRRSV-1 strain circulating from Batches 0 to 5. This particular strain presented distinct variants, namely a monophyletic branch that has a number of marker mutations that distinguish them: 1α, which was detected as the solely variant in Batches 0 and 1, and 1β, which was present from Batches 2 to 5. Both variants shared >99.5 per cent of nucleotide similarity (Supplementary Table S5) but were distinguishable by certain marker mutations and deletions. Subsequently, during the nursery period in Batch 5, a new strain (referred to as strain 2) was identified in some nursery samples, but not in the farrowing units. This strain 2 showed only 83 per cent nucleotide similarity to the previously observed viral variants 1α and 1β (Supplementary Table S5), and eventually became the dominant strain, persisting as the sole strain detected until the end of the study (Batch 7). The analysis of ORF5 sequences from all RT-qPCR positive samples with *C*_t_ values <32 (Supplementary Fig. S1) confirmed the absence of other variants or strains. Furthermore, when comparing with sequences from commercially available attenuated vaccines, the variants found in our study did not correspond to the vaccine used on the farm, nor to any other available vaccines (Supplementary Figs S2 and S3, and Supplementary Table S6).

**Figure 4.**
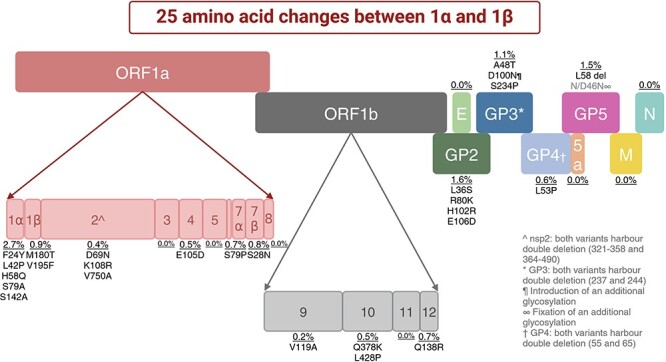
Distribution of non-synonymous mutations and deletions in variant 1β compared to variant 1α. The figure shows the non-synonymous amino acid changes across the viral genome in all sequenced isolates for variant 1β (synapomorphic traits) that were absent in the consensus sequences of 1α, with indication of the protein affected and, for ORF1a and ORF1b, the non-structural proteins (nsp) involved. Amino acids are represented with a single-letter code. The first letter indicates the predominating amino acid in variant 1α, the number the position in the protein, and the second letter indicates the amino acid in variant 1β. Created with BioRender.com.

The comparison of amino acid composition of viral proteins between variants 1α and 1β, inferred from their consensus nucleotide sequences, revealed that they differed by only twenty-five positions across the whole genome for all examined isolates (Fig. 4 and Supplementary Table S7). Of note, these mutations were not randomly distributed. For example, nsp1α (180 aa), comprising only 3.8 per cent of the genome’s coding regions, accumulated five non-synonymous mutations, representing 20 per cent of the total number of non-synonymous mutations in the entire genome. Similarly, GP2 (249 aa, 5.3 per cent of the coding regions) and GP3 (263 aa, 5.5 per cent of the coding regions) exhibited four and three non-synonymous mutations, respectively, with GP3 also gaining an additional glycosylation site at position 100. In contrast, nsp2 of variant 1β (17.8 per cent of the coding regions of the genome and the largest protein in PRRSV) only harboured three non-synonymous mutations (12 per cent of the total number of this type of mutations) compared to 1α. To note, variant 1β had a deletion at position 58 of GP5, downstream of the neutralization epitope in this protein, and the fixation of a glycosylation site at position 46, which was also seen in some isolates of 1α. These findings suggest that the selection pressures on different viral proteins were different.

Interestingly, both variants 1α and 1β presented two deletions in GP3, at positions 237 and 244 (compared to Lelystad virus), and a double deletion in GP4, at positions 55 and 65, which correspond to the neutralization epitope described by Costers and Lefebvre et al. (2010a).

Given the rapid replacement of variant 1α by variant 1β and the presence of relatively high-fixed mutations (25), we examined whether different mutations or groups of mutations co-occurred or independently appeared in different animals. Analysis of viral quasi-species in 1α isolates for nsp1 and GP2 revealed the existence of all fixed mutations in 1β, although the majority were at low frequencies (<5 per cent of reads) (Table 2). However, high frequencies of F24Y and L42P in nsp1α seemed to be linked one to another in some animals but not to other mutations. Notably, higher mutation frequencies in nsp1α were not related to increased frequency of the mutations in GP2 or the new glycosylation in GP5. These findings suggest that the selection of mutations in different proteins of the virus likely did not occur in a single animal, and the generation of the predominant set of β mutations may not have been the outcome of gradual selection from a single variant.

**Table 2.** Frequency of amino acid changes fixed in variant 1β within the quasi-species of variant 1α (*n* = 20 animals). The table shows the frequency, expressed over 1, of the indicated mutations for nsp1α and GP2. The last column shows the frequency of an additional glycosylation in GP5 that was already pre-eminent in some 1α isolates as a reference.

		Viral protein									
		nsp1α					GP2				GP5
	Mutation	F24Y	L42P	H58Q	S79A	S142A	L36S	R80K	H102R	E106D	N/D46N
Batch-Animal	B0-700-8w	0.13	0.16	0.06	0.03	0.05	0.04	0.03	0.04	0.01	0.98
	B0-702-8w	0.09	0.1	0.95	0.02	0.06	0.04	0.04	0.04	0.01	0.06
	B0-704-8w	0.1	0.16	0.04	0.05	0.03	0.05	0.04	0.02	0.01	0.05
	B0-712-8w	0.12	0.16	0.04	0.04	0.05	0.03	0.03	0.02	0.01	0.03
	B0-745-8w	0.14	0.15	0.04	0.04	0.05	0.03	0.02	0.02	0.01	0.03
	B0-749-8w	0	0	0	0	0.02	0.01	0	0.01	0	0.91
	B0-750-8w	0.06	0.1	0.03	0.03	0.03	0.03	0.03	0.04	0.02	0.02
	B1-666-2w	0.01	0.04	0.02	0.01	0.01	0.05	0.03	0.01	0.01	1
	B1-666-4w	0.01	0.07	0.04	0.04	0.02	0.02	0.02	0.02	0.02	1
	B1-666-6w	0.04	0.01	0.01	0.01	0.01	0.05	0.02	0.02	0.01	1
	B1-867-9w	0.01	0.06	0.02	0.05	0.02	0.02	0.02	0.02	0.02	1
	B1-876-2w	0.02	0.07	0.03	0.03	0.03	0.04	0.03	0.04	0.03	0.02
	B1-890-6w	0.02	1	0.02	0.03	0.02	0.03	0.04	0.02	0.01	0.05
	B1-900-9w	0.03	0.06	0.02	0.02	0.03	0.02	0.01	0.01	0	0.02
	B1-909-6w	0.05	0.04	0.03	0.02	0	0.02	0.02	0.02	0.01	0.02
	B1-923-9w	0.04	0.05	0.02	0.04	0.04	0.06	0.05	0.04	0.01	0.06
	B1-926-6w	0	0	0	0	0	0.01	0	0	0	0.04
	B1-957-9w	0.03	0.05	0.02	0.02	0.01	0.01	0.02	0.03	0.02	0.02
	B1-989-6w	0	0	0.01	0.01	0	0.02	0	0.01	0	0
	B1-996-4w	0.03	0.07	0.05	0.03	0.03	0.06	0.06	0.08	0.05	0.98

Next, a recombination analysis was conducted to determine if recombination could be detected in the obtained sequences. The results (Supplementary Figs S4 and S5) showed that recombination occurred between variants 1α and 1β and affected a segment of 129 nucleotides in a highly variable region of the nsp2 (positions 2,235–2,958 according to the alignment with PRRSV-1 prototype Lelystad; Accession Number NC_043487). This suggests that both variants probably co-existed within the farm for some time and co-infected some individuals.

### 3.3 Serological analyses

**Figure 5.**
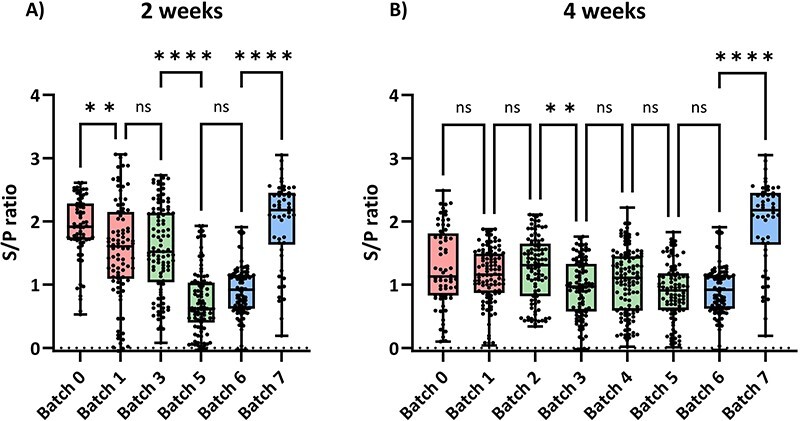
Antibody levels against the viral nucleocapsid protein expressed as *S*/*P* ratios as determined in ELISA. Each dot represents an individual. (A) Animals examined at two weeks of age (Batches 2 and 4 were not examined at that age). (B) Animals examined at four weeks of age. ns = non-significant differences. *P < 0.05; **P < 0.01; ***P < 0.001; ****P < 0.0001.

The presence of maternally derived antibodies was determined at two and four weeks of age by ELISA (Fig. 5). The results showed that significant differences can be observed for two-week-old piglets between batches where the same variant/strain was circulating. Differences were not observed at four weeks of age. Moreover, *S*/*P* values were not correlated with VTE frequencies nor with the incidence in the farrowing units (data not shown). Supplementary Fig. S6 shows the *S*/*P* values for piglets of six and nine weeks of age.

**Figure 6.**
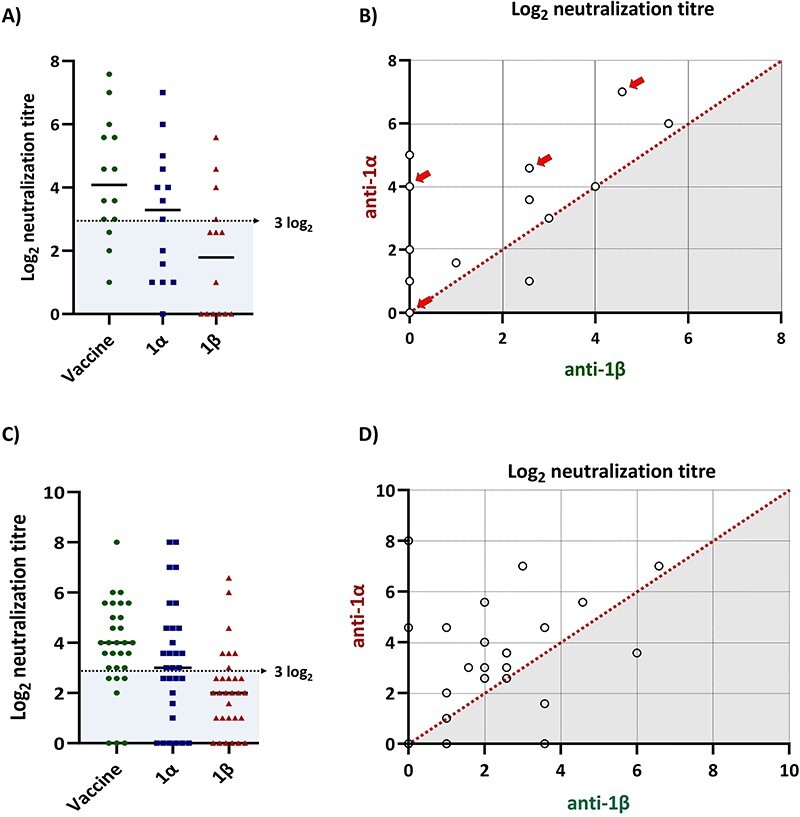
Neutralizing antibody titres in sows and piglets against the vaccine strain and the variants 1α and 1β. (A) Distribution of neutralization titres of sows from Batch 1 against the vaccine virus, the variant 1α, and variant 1β. The shadowed area shows the individuals whose result was below 3 log_2_ for each tested viral isolate. (B) Comparison of neutralization titres obtained for each sow from Batch 1 with variant 1α and variant 1β. The diagonal (dotted line) represents the line of identity for both tests. Arrows indicate sows that gave birth to viraemic animals. (C) Distribution of neutralization titres of piglets at two weeks of age from Batch 1 against the vaccine virus, the variant 1α, and variant 1β. The shadowed area shows the individuals whose result was below 3 log_2_ for each tested viral isolate. (D) Comparison of neutralization titres obtained for each piglet at two weeks of age from Batch 1 with variant 1α and variant 1β. The diagonal (dotted line) represents the line of identity for both tests.

Since sera from sows and piglets present in Batch 1 were available, namely preceding the emergence of variant 1β, it was possible to test their capacity for neutralizing 1β (Fig. 6). In ten sows (71 per cent), the titres against 1α were equal or higher than the titres obtained for 1β neutralization, with differences ranging from 1 to 5 log_2_. Remarkably, the sera of six sows were completely devoid of any neutralizing capacity against 1β. Similarly, when two-week-old piglets were analysed, most of them (24/31; 77.4 per cent) showed higher titres against 1α compared to 1β, with differences ranging from 0.5 to 3.5 log_2_.

Interestingly, within the group of fourteen sows, four had transmitted vertically the infection to their offspring. Of these, three had neutralization titres against 1α (4.6–7.0 log_2_) and the fourth sow was negative against 1α or 1β despite having a titre >5 log_2_ against the vaccine strain (data not shown).

Vaccine virus was used as a reference in the VNT. The results showed that titres against vaccine virus and 1α in both sows and piglets were not significantly different but were higher than the titres against 1β (*P* < 0.05). Levels of neutralizing antibodies against the vaccine in the sows had limited predictive value for the results against 1α (*R*^2^ = 0.3897, *P* = 0.017) and even less against 1β (*R*^2^ = 0.2752, *P* = 0.054) (Supplementary Fig. S7).

In Batches 5, 6, and 7, two-week-old pigs were assessed for levels of neutralizing antibodies. All animals analysed after Batch 5 had no detectable antibodies against 1α or 1β, suggesting that memory of those infections may have faded out from the farm (data not shown).

When nine-week-old piglets were examined in the VNT, only animals from Batch 1 (6/15; 40 per cent) had titres ≥2 log_2_ against variant 1α, the one circulating in that batch (range 2–8 log_2_). No neutralizing capacity was detected against any of the examined viruses in nine-week-old pigs from Batches 2 to 7 (data not shown).

### 3.4 Replication kinetics of variants 1α and 1β in PAM

**Figure 7.**
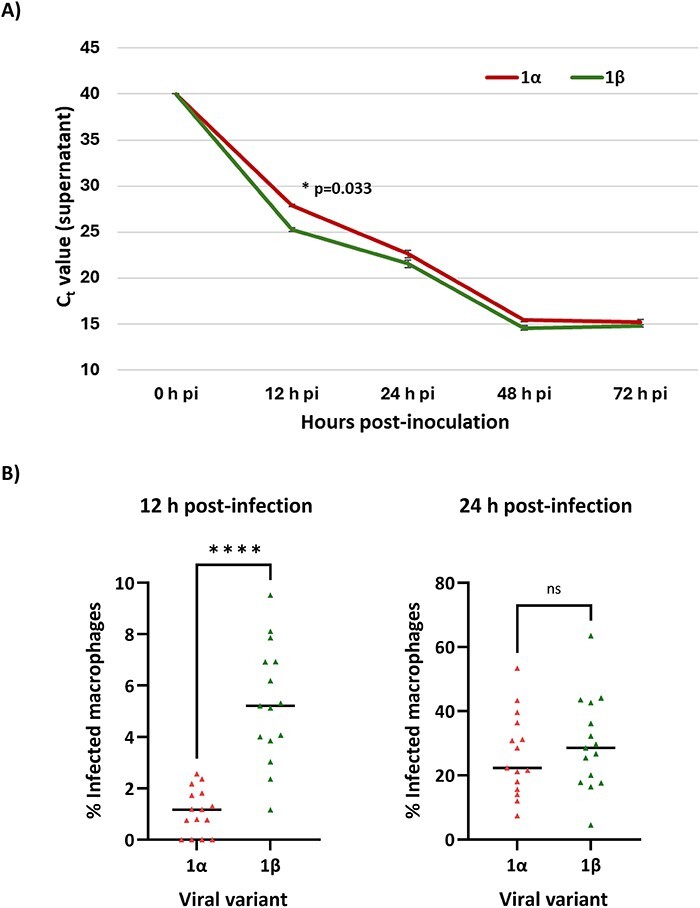
Results of the replication kinetics experiment for variants 1α and 1β. (A) *C*_t_ values for the cell culture supernatants of PAM infected with variant 1α (red) and 1β (green) at MOI 0.1. (B) Proportion of PAM labelling positive for PRRSV in the same cultures after 12 and 24 h of incubation. ns = non-significant differences. *P < 0.05; **P < 0.01; ***P < 0.001; ****P < 0.0001.

The emergence of variant 1β coincided with a decrease in the average *C*_t_ values of infected nursery pigs compared to *C*_t_ values when 1α was circulating, indicating an increase in the viral loads. To determine whether these differences were due to varying replication efficiencies, a replication kinetics experiment was performed. Figure 7 illustrates the replication kinetics of 1α and 1β in PAM over 72 h. Notably, a significantly (*P* < 0.05) lower *C*_t_ value (2.2 vs 2.9 log_10_) was observed at 12 h post-infection in supernatants from 1β-inoculated cultures (Fig. 7A). This was paralleled by a higher proportion of infected PAM at the same time point (Fig. 7B). But the difference in *C*_t_ values and infection rates between the two variants did not persist at later time points (Fig. 7A, B). Our results hint that the superior replication capability of 1β may have potentially contributed to the increased viral loads observed in nursery pigs.

### 3.5 Attachment of variants 1α and 1β on PAM

**Figure 8.**
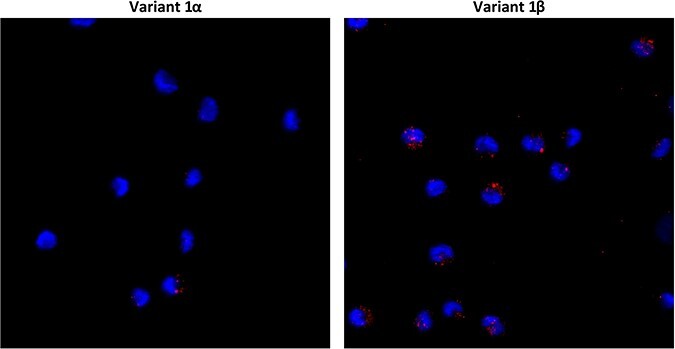
Attachment of variants 1α and 1β to PAM determined by confocal microscopy. The images show confocal microscopy *z*-projections of the attachment of variants 1α and 1β on PAM. PRRSV N protein and PAM nuclei were stained differently (red and blue, respectively).

The findings of the replication kinetic experiments suggested that differences between variants 1α and 1β might stem from disparities in the initial stages of the viral replication cycle. For this purpose, an attachment experiment on PAM was performed. Visually, the results showed more 1β viral particles attaching on PAM compared to 1α (Fig. 8), suggesting that the enhanced attachment capability of 1β could be a contributing factor to its increased higher viral yields at early replication times.

### 3.6 Inhibition of poly I:C induced IFN-α and TNF-α

Next, we explored the possibility that the emergence of variant 1β could have resulted from an enhanced capability to inhibit innate antiviral responses. For this purpose, the ability of both variants to inhibit IFN-α and TNF-α responses was examined using PAM cultures. When PAMs were exposed to poly I:C either previously or simultaneously with the virus, the release of IFN-α was reduced by more than 80 per cent for both strains. Inoculating PAM with either the variant 1α or 1β prior to the addition of poly I:C led to a significant reduction of TNF-α levels, with no significant differences observed between the variants (Supplementary Fig. S8).

## 4. Discussion

Control and eradication of PRRSV stands as one of the utmost priorities for endemically infected farms. The strategies to achieve those goals are based on four pillars: monitoring of the infection, immunization of the herd, management of the pig flow, and biosecurity measures. However, despite the implementation of highly stringent protocols, certain farms are very difficult to stabilize, with infected animals being continuously found in the farrowing units or nurseries. For instance, Trevisan et al. (2019) reported that even following herd vaccination and interruption of replacement gilts entry, more than six months of closure were needed to confirm the complete cessation of viral circulation in 50 per cent of the cases. Moreover, some farms required over a year of closure, while others consistently failed to achieve a negative status.

Certainly, there are several factors that contribute to the persistence of the virus within the herd, which are well known and are shared by many communicable diseases. These include the introduction of replacement gilts without prior quarantine and vaccination, the mingling of susceptible and infected animals, and inadequate management practices that promote transmission through fomites, among others. Nonetheless, the role of the viral evolution in that context remains a scarcely explored area. The objective of the present work was to gain insight on how the virus evolves and persists within a farm under a strong pressure of vaccination.

The studied farm had been experiencing PRRSV circulation for years despite all efforts aimed at controlling the infection. At the commencement of the study, the farm was unstable, as evidenced by the detection of VTE occurring at birth. Somewhat surprisingly, the infection spread relatively slowly, with only 60 per cent of the pigs being infected by the end of the nursery phase. Considering that maternally derived antibodies usually wane around the fourth or fifth week of age (Andraud et al. 2018) and that piglets had not been vaccinated, a faster dissemination of the infection would have been anticipated. Subsequently, in the next batch, this trend persisted, with only 36 per cent of piglets infected in the nurseries. From then on, there were sharp increases in the incidence followed by a subsequent decline. The analysis of the obtained viral sequences shed light on the reasons behind this observed behaviour.

The phylogenetic analysis showed that during Batches 0–5, a single viral strain, designated as strain 1, was circulating. Also, it was observed that during the sampling period of Batch 5, a distinct strain (83 per cent nucleotide identity), referred to as strain 2, was introduced into the farm from an external source. Upon closer examination of the results, it was also observed that strain 1 actually existed as two distinct variants, designated as 1α and 1β, which differed less than 0.5 per cent of the nucleotide sequence. Notably, variant 1α was exclusively detected in Batches 0 and 1 being subsequently replaced completely by variant 1β within a matter of a few weeks. This suggested that variant 1β had probably certain features that made it fitter for transmission within the context of a highly vaccinated farm.

The analysis of the *C*_t_ values of the animals infected in nurseries (lacking passive immunity) showed that the emergence of variant 1β correlated with a significant reduction in the average *C*_t_ values of infected nursery pigs. In other words, the viral loads of the grower pigs infected with 1β were higher compared to those infected with 1α in the previous batch, thereby suggesting again that variant 1β exhibited greater fitness than 1α within that particular context. Interestingly, in subsequent samplings, the average *C*_t_ values increased, indicating a decrease in viral loads. The introduction of strain 2 once again appears to have resulted in increased viral loads with a similar pattern as described earlier.

Moreover, there seems to be a strong correlation between the average *C*_t_ of infected growers and the incidence in nurseries. Considering these findings collectively, it is plausible to hypothesize that variant 1β was fitter than 1α to replicate in pigs, consequently spreading faster in the nurseries. However, the subsequent decrease in the viral loads suggests that as the virus was transmitted to a larger number of animals over time, some form of selective constraint tended to limit viral replication within the studied context. It is unlikely that this factor was immunity in the piglets since, in fact, most of them were infected when they had already lost maternally derived antibodies. An alternative explanation could be that when incidence increases, more animals are infected per time unit, leading to a higher number of animals reaching the peak of viraemia at the same time. In contrast, with lower incidences, the course of infection may not be synchronized.

One interesting finding in our study was that the emergence of variant 1β was not significantly related to an increase in the VTE or with the viral transmission in the farrowing units. The most reasonable explanation for this phenomenon is that most of the sows would have some level of immunity (either neutralizing antibodies or cell-mediated immunity) due to prior vaccinations or contact with strain 1α. Similarly, a high proportion of two-week-old piglets in Batches 0–5 had biologically relevant titres of neutralizing antibodies, which would certainly impede the transmission of the virus in the farrowing units. The introduction of strain 2 did result in an increase in VTE as expected, given its genetic distance to strain 1.

Once established that the emergence of a new variant, or the lateral introduction of a new strain, resulted in the rapid displacement of the former circulating variant, together with an increase in the incidence in the nurseries, the subsequent step entailed attempting to understand the mechanisms leading to this phenomenon. The comparison of the consensus sequences of 1α and 1β revealed that non-synonymous mutations were not randomly distributed, but rather accumulated in nsp1 (α and β), GP2, and GP3, and included one deletion in GP5, immediately following the known neutralization epitope in that protein. In arteriviruses, nsp1 has been reported to control the quantity of minus-strand templates for mRNA synthesis, thereby regulating replication (Nedialkova, Gorbalenya, and Snijder 2010), but it also has an important role in inhibiting type I interferon responses in PRRSV-infected cells (Han and Yoo 2014). GP2 and GP3, together with GP4, form a heterotrimer that interacts with CD163, the essential receptor for PRRSV (Calvert et al. 2007; Das et al. 2010; Van Gorp et al. 2010; Yu et al. 2020). Besides, GP5 interacts with porcine sialoadhesin, a co-receptor of PRRSV (Van Breedam et al. 2010), and it has been reported that GP5 may also interact with CD163 (Yu et al. 2020). Neutralizing antibodies against GP3, GP4, and GP5 have been documented in various studies (Vanhee et al. 2011), although contradictory reports also exist (Van Breedam et al. 2011; Li and Murtaugh 2012). In addition, the neutralization epitope in GP4 is known to be very variable and may serve as a driving force in the selection of neutralization escape mutants (Costers et al. 2010b). Considering this background information and the epidemiological data, we hypothesized that 1β could potentially be an escape mutant of 1α with enhanced replication capability.

To evaluate that hypothesis, we assessed whether the sera of animals (sows and their piglets) that were present while 1α was circulating and before the emergence of 1β had or not the same capability to neutralize both variants. The results indicated that, for most of the tested animals, neutralization titres were higher against 1α than against 1β. This supports the hypothesis that 1β was an escape mutant able to evade neutralization by the anti-1α antibodies. By employing a cut-off value of 3 log_2_ to consider the existence of homologous protection produced by neutralizing antibodies (Osorio et al. 2002; Lopez et al. 2007), it was observed that three out of four sows, or a similar proportion of their offspring in the farrowing units, would not be protected against infection by 1β. Interestingly, this included some sows with titres as high as 5 log_2_ against 1α. The most likely explanation for these differences is that 1β was indeed a neutralization escape mutant, potentially due to the mutations in the structural glycoproteins that are known to induce neutralizing antibodies. Moreover, these results also pointed specific amino acid positions in GP2, GP3, GP4, and GP5 that could be important targets for modifying the neutralizing characteristics of PRRSV-1 strains. Further laboratory investigations could delve into this topic. Besides, it is worth noting that both variants harboured double deletions in GP3 and GP4, with the latter encompassing the known neutralization epitope reported by Costers and Lefebvre et al. (2010a). Additionally, 1β introduced a deletion in GP5. To our knowledge, this is the first report of a PRRSV-1 isolate presenting simultaneously multiple deletions in all those structural glycoproteins.

Regarding strain 2, the dissemination within the farm can be explained simply by the genetic distance and the lack of specific neutralizing antibodies in the sows. It is well known that, in most cases, the neutralizing antibodies induced by one PRRSV-1 strain are little reactive against other genetically distant counterparts (Martínez-Lobo et al. 2011).

As previously mentioned, the rapid spread of variant 1β and the increased viral loads suggested that this variant had potentially a better replication fitness in comparison to variant 1α. In the replication kinetic experiments, 1β produced a higher yield of virus and a higher proportion of infected cells within the initial 12-h period of incubation, but not in later time intervals. This fact indicates that the observed differences could possibly be attributed to better attachment or internalization capabilities. Accordingly, an experiment was set up to test this phenomenon. The results showed that 1β appeared to exhibit a higher degree of attachment on PAM compared to 1α, probably resulting in increased replication in the cells, most likely due to the internalization of a larger number of viral particles. Obviously, this would confer an advantageous feature to 1β. It is tempting to postulate that this increased ability to infect PAM and the resulting increased viral loads would culminate in an enhanced viral shedding, thus accelerating the transmission rate of 1β. The underlying mechanism behind this would point again to GP5, as it interacts with porcine sialoadhesin on the surface of PAM (Van Breedam et al. 2010) and possibly with CD163, and GP2 and GP3, due to their interaction with CD163 (Yu et al. 2020). Once again, the observed mutations and deletions may indicate the presence of critical residues within those proteins.

We finally examined whether 1β had a higher capability to regulate the production of innate antiviral cytokines (IFN-α and TNF-α) in inoculated PAM. The results indicated that although both strains strongly downregulated the IFN-α and TNF-α responses following TLR-3 stimulation, no statistically significant differences were observed. This suggests that this mechanism was not implicated in the emergence of variant 1β.

An intriguing question is how the variant 1β was originated. Several facts must be considered: first, the mutations fixed in the 1β variant were already present as minor variants within the 1α quasi-species of some individuals; second, within an individual, the increase in one mutation was not related to the increase in the frequency of another mutation, except within nsp1α; third, there is evidence of recombination between 1α and 1β; and fourth, the replacement of 1α was extremely fast and it disappeared from the population in just few weeks. Taken together, it seems plausible that at one point, several 1α subvariants, each one harbouring one or more advantageous mutations, may have undergone recombination to produce the founder 1β variant. The alternative notion of a gradual selection of up to twenty-five mutations to produce 1β is difficult to reconcile with these observed facts.

At this point, it is possible to draw a picture of how farms may persist endemically infected over prolonged periods of time. Our study showed that, besides the occurrence of lateral introductions of the virus, a combination of evolutionary events may result in the emergence of variants that accumulate several mechanisms to gain fitness in an immune, or partially immune, population. These mechanisms include the evasion of neutralizing antibodies and enhanced capability to infect PAM. Whether these two mechanisms are related to the same mutations or not cannot be definitively inferred from the present study. However, this can be experimentally evaluated in further studies.

Nevertheless, several unresolved inquires remain. The first one pertains to the origin of variant 1β, whether it emerged in sows or piglets. Another question that deserves further investigation is why after the emergence of a variant (or the introduction of a new strain) and the initial increase in the incidence, there was a subsequent decrease. Unfortunately, we are unable to postulate an explanation based on evidence for these facts.

The current study highlights the plasticity of PRRSV and the several mechanisms that the virus can use to persist in the population despite intensive vaccination protocols. Moreover, this also reveals that as long as the virus continues to circulate in the population, it will be very difficult to avoid the emergence of novel variants with enhanced capability for infecting pigs. Consequently, this points towards the need for developing newer and more efficacious vaccines, as well as to implement eradication programmes with all available resources.

## Supplementary Material

veae041_Supp

## Data Availability

The datasets are available in GenBank and accession numbers can be found in the main text or the supplementary materials.
