# Supplementary material for: Selection of viral variants with enhanced transmission and reduced neutralization susceptibility alongside lateral introductions may explain the persistence of porcine reproductive and respiratory syndrome virus in vaccinated breeding herds
Source: Virus Evol. 2024 May 17;10(1):veae041. doi: 10.1093/ve/veae041 (PMC11137674; doi:10.1093/ve/veae041)

SUPPLEMENTARY MATERIAL

Selection of viral variants with enhanced transmission and reduced neutralization susceptibility alongside lateral introductions may explain the persistence of porcine reproductive and respiratory syndrome virus in vaccinated breeding herds.

Hepzibar Clilverd, Yanli Li, Gerard Martín-Valls, Laia Aguirre, Marga Martín, Martí Cortey, Enric Mateu

Supplementary Table 1. List of oligonucleotide primers (F: forward primer, R: reverse primer) used for amplifying PRRSV-1 ORF5 and the size of the amplicon. p.2

Supplementary Table 2. PRRSV-1 complete genome sequences retrieved from GenBank for phylogenetic analysis. p.3

Supplementary Table 3. Amino acid mutations resulting from the adaptation of PRRSV-1 isolates to MARC145-cells. p.4

Supplementary Table 4. Complementary data on the followed pig batches with indication of the results used for the calculation of the incidences and the incidences at each timepoint. p.5

Supplementary Table 5. Similarities (p-distance ± standard error) within and between variant 1α, 1β, and strain 2. p.6

Supplementary Table 6. Similarities (p-distance) between the circulating viruses in this study and the reference PRRSV-1 strains. p.7

Supplementary Table 7. Amino acid comparison of PRRSV-1 sequences variants 1α, 1β, and strain 2. p.8

Supplementary Figure 1. Bayesian tree showing the phylogenetic grouping of the ORF5 sequences obtained in this study. p.13

Supplementary Figure 2. Bayesian phylogenetic tree based on whole genome sequences of PRRSV-1. p.14

Supplementary Figure 3. Bayesian phylogenetic tree based on ORF5 sequences of PRRSV-1. p.15

Supplementary Figure 4. Genome recombination event between variants 1α and 1β. p.16

Supplementary Figure 5. Example of the genetic distance plots for a recombination event. p. 17

Supplementary Figure 6. Antibody levels of six- and nine-week-old piglets against the viral nucleocapsid protein expressed as S/P ratios as determined in ELISA. p.18

Supplementary Figure 7. Neutralizing antibody titres in sows from Batch 1 against the variants 1α and 1β and the vaccine virus. p.19

Supplementary Figure 8. Inhibition of the levels of poly I:C induced IFN-α and TNF-α in PAM. p.20

Supplementary Table 1. List of oligonucleotide primers (F: forward primer, R: reverse primer) used for amplifying PRRSV-1 ORF5 and the size of the amplicon.

Gene	Sequence
**ORF5**	
**Forward L1F**	5′- TGAGGTGGGCTACAACCATT -3′
**Reverse C1**	5′- GCGTGACACCTTAAGGGC -3′
**Reverse C6**	5′- GCGACACCTTAAGGGCATATATCA -3′

Supplementary Table 2. PRRSV-1 complete genome sequences retrieved from GenBank for phylogenetic analysis. Lelystad virus is the prototype for PRRSV-1. Contemporary strains in the farm's geographical zone (underlined) and the five licensed vaccines in Spain (in red) are included. These PRRSV-1 strains were employed for comparative analyses in Supplementary Table 5, Figures 2, and 3. Adapted from Clilverd et al., (2023).

Accession Number	Strain ID	Country	Year
NC043487	***Lelystad virus***	Netherlands	1993
KF203132	*Olot/91*	Spain	1991
JF276431	*CReSA3262*	Spain	1992
KX249748	<u>*CReSA3*</u>	Spain	2013
KX249755	<u>*CReSA228*</u>	Spain	2013
KX249756	<u>*CReSA261*</u>	Spain	2013
KX249753	<u>*CReSA100*</u>	Spain	2014
OP688199	<u>*B1-522-4w*</u>	Spain	2017
OP688221	<u>*B3-785-9w*</u>	Spain	2018
OP822977	<u>*Nu4a*</u>	Spain	2021
OP822964	<u>*M3*</u>	Spain	2021
OP822963	<u>*N5*</u>	Spain	2021
OP822973	<u>*Nu1*</u>	Spain	2021
OM893828	<u>*R1*</u>	Spain	2021
OM893829	<u>*R2*</u>	Spain	2021
JF276435	*CReSA3267*	Portugal	2006
KT326148	*AUT13-883*	Austria	2013
JF802085	*Lena*	Belarus	2007
KP889243	*SU1-Bel*	Belarus	2010
GU737264	*07V063*	Belgium	2007
KT159248	*13V091*	Belgium	2013
KT159249	*13V117*	Belgium	2013
GU047344	*BJEU06-1*	China	2006
MN927227	*HeB3*	China	2018
KC862567	*DK-2011-0511-14*	Denmark	2011
KY767026	*FR-2014-56-11-1*	France	2014
MH018883	*FR-2016-56-11-1*	France	2016
MH463457	*HU19401*	Hungary	2016
MF346695	*PR40-2014*	Italy	2014
KX668221	*WestSib13*	Russia	2013
KY434183	*CBNU0495*	South Korea	2016
GU067771	***Unistrain®PRRS***	-	-
KJ127878	***Porcilis® PRRS***	-	-
LQ787782	***Suvaxyn® PRRS***	-	-
GQ461593	***PYRSVAC-183®***	-	-
KT988004	***PRRSFlex®EU***	-	-

Supplementary Table 3. Amino acid mutations resulting from the adaptation of PRRSV-1 isolates to MARC145-cells. The table displays the accumulated non-synonymous amino acid mutations across the genome resulting from the adaptation after four cellular passages of an isolate of variants 1α and 1β and strain 2 to MARC145-cells. Nucleotide position correspond to the alignment with the prototype PRRSV-1 strain Lelystad (LV; NC_043487). Amino acids are represented with a one-letter code. The first letter indicates the amino acid in the isolate, the number the position in the protein, and the second letter the amino acid in isolate adapted to MARC-145 cells.

Strain/Virus	Protein	Amino acid mutation
**Variant 1α**	**nsp7b**	S51P
	**nsp9**	F533L
	**nsp10**	R429G
	**GP2**	V170A
	**GP4**	I67Del
**Variant 1β**	**nsp2**	H499Q
		S506R
		I509L
		P518Q
**Strain 2**	**nsp2**	Y226C
	**nsp10**	P281L

Supplementary Table 4. Complementary data on the followed pig batches with indication of the results used for the calculation of the incidences and the incidences at each timepoint.

Batch	Susceptible piglets	Animals with missing data	PRRSV PCR+ UC	PRRSV PCR+ at 2 woa	PRRSV PCR+ at 3 or 4 woa	PRRSV PCR+ at 6 woa	PRRSV PCR+ (+seropositive) at 9 woa	PRRSV PCR+ in farrowing units	PRRSV PCR+ in nurseries (+seropositive)	Incidence (PCR+) at 2 woa	Incidence (PCR+) at 3 or 4 woa	Incidence (PCR+) at 6 woa	Incidence (PCR+) at 9 woa (+seropositive)
**0**	62	7	3	1	0	3	33 (49)	1	36 (52)	1.6%	0.0%	4.9%	56.9% (84.5%)
**1**	68	37	8	4	3	4	15 (26)	7	19 (30)	5.9%	4.7%	6.6%	26.3% (45.6%)
**2**	60	38	0	N.A.	1	0	52 (55)	1	52 (55)	N.A.	1.7%	0.0%	88.1% (93.22%)
**3**	95	1	1	3	0	2	52 (66)	3	54 (68)	3.2%	0.0%	2.2%	57.8% (73.3%)
**4**	63	52	0	N.A.	1	1	9 (20)	1	10 (21)	N.A.	1.6%	1.6%	14.8% (32.8%)
**5**	76	14	5	5	3	22	44 (45)	8	66 (67)	6.7%	4.2%	32.4%	95.7% (97.8%)
**6**	92	3	0	N.A.	1	15	45 (54)	2	60 (69)	N.A.	1.1%	16.5%	59.2% (71.1%)
**7**[a]	N.A.	N.A.	2	N.A.	21	10	11	21	21 (9)	N.A.	42% [a]	50% [a]	50% [a] (100%)
**Totals/ Averages**										4.3%±2.3%	2.0%±2.0%	7.9%±12.2%	56.6%±32.2% (71.21%±26.5)

[a]= In this case a cross-sectional sampling was performed. Data represent prevalence not incidence; it has not been included in the total calculated average. [$]= Differences were calculated with regards to the previous batch. N.A.= Does not apply. PCR+= PCR positive. woa= weeks of age.

Supplementary Table 5. Similarities (p-distance ± standard error) within and between variant 1α, 1β, and strain 2. The table presents the average nucleotide differences within and between the different variants of PRRSV-1 whole genome consensus sequences and individual segments of the viral genome. nsp = non-structural protein.

Segment	Variant 1α	Variant 1β	Strain 2	1α vs. 1β	1α vs. 2	1β vs. 2
**Genome**	0.0034±0.0003	0.0029±0.0002	0.0092±0.0005	0.0046±0.0053	0.1676±0.0031	0.1687±0.0031
**ORF1a**						
**nsp1α**	0.0034±0.0013	0.0034±0.0012	0.0134±0.0028	0.0014±0.0047	0.1355±0.0145	0.1276±0.0140
**nsp1β**	0.0051±0.0012	0.0020±0.0005	0.0080±0.0019	0.0066±0.0031	0.2133±0.0161	0.2215±0.0164
**nsp2**	0.0046±0.0007	0.0048±0.0008	0.0129±0.0012	0.0035±0.0010	0.2023±0.0077	0.2021±0.0077
**nsp3**	0.0026±0.0008	0.0025±0.0008	0.0110±0.0020	0.0062±0.0026	0.1812±0.0127	0.1860±0.0129
**nsp4**	0.0027±0.0008	0.0025±0.0010	0.0083±0.0022	0.0035±0.0022	0.1792±0.0156	0.1783±0.0157
**nsp5**	0.0043±0.0016	0.0025±0.0010	0.0050±0.0017	0.0020±0.0013	0.1751±0.0170	0.1765±0.0170
**nsp6**	0.0021±0.0020	0.0000±0.0000	0.0096±0.0093	0.0000±0.0000	0.1688±0.0547	0.1688±0.0547
**nsp7a**	0.0020±0.0008	0.0038±0.0014	0.0076±0.0023	0.0053±0.0033	0.1543±0.0173	0.1553±0.0171
**nsp7b**	0.0063±0.0024	0.0035±0.0015	0.0112±0.0030	0.0048±0.0029	0.1748±0.0199	0.1713±0.0200
**nsp8**	0.0029±0.0028	0.0004±0.0004	0.0152±0.0054	0.0038±0.0039	0.1541±0.0303	0.1537±0.0303
**ORF1b**						
**nsp9**	0.0022±0.0006	0.0019±0.0005	0.0052±0.0009	0.0027±0.0011	0.1451±0.0079	0.1457±0.0079
**nsp10**	0.0036±0.0009	0.0027±0.0007	0.0074±0.0014	0.0047±0.0018	0.1641±0.0099	0.1662±0.0100
**nsp11**	0.0016±0.0006	0.0022±0.0009	0.0078±0.0019	0.0005±0.0005	0.1562±0.0139	0.1564±0.0138
**nsp12**	0.0032±0.0015	0.0032±0.0012	0.0094±0.0027	0.0068±0.0035	0.1727±0.0168	0.1762±0.0169
**ORF2a**	0.0024±0.0009	0.0009±0.0003	0.0110±0.0023	0.0085±0.0032	0.1456±0.0128	0.1514±0.0131
**ORF2b**	0.0005±0.0005	0.0017±0.0010	0.0094±0.0036	0.0047±0.0047	0.0799±0.0187	0.0838±0.0192
**ORF3**	0.0051±0.0012	0.0033±0.0009	0.0009±0.0019	0.0059±0.0024	0.1611±0.0128	0.1602±0.0128
**ORF4**	0.0014±0.0007	0.0029±0.0010	0.0090±0.0023	0.0053±0.0030	0.1377±0.0151	0.1406±0.0151
**ORF5a**	0.0060±0.0044	0.0034±0.0020	0.0087±0.0042	0.0028±0.0026	0.1382±0.0285	0.1372±0.0288
**ORF5**	0.0039±0.0010	0.0032±0.0009	0.0114±0.0023	0.0043±0.0024	0.1760±0.0152	0.1780±0.0154
**ORF6**	0.0010±0.0004	0.0021±0.0011	0.0145±0.0023	0.0045±0.0027	0.1157±0.0139	0.1180±0.0139
**ORF7**	0.0028±0.0015	0.0007±0.0004	0.0052±0.0021	0.0061±0.0038	0.1070±0.0149	0.1065±0.0150

Supplementary Table 6. Similarities (p-distance) between the circulating viruses in this study and the reference PRRSV-1 strains. The table depicts the nucleotide mean differences among the examined variants of PRRSV-1 whole genome consensus and ORF5 sequences and the sequences obtained from GenBank.

	**Genome**			**ORF5**		
**PRRSV-1 strain**	1α	1β	2	1α	1β	2
***Lelystad virus*-NC043487**	0,1314	0,1361	0,1278	0,1486	0,1488	0,1151
Olot/91-KF203132	0,1285	0,1336	0,1376	0,1386	0,1388	0,1220
CReSA3-KX249748	0,1605	0,1632	0,1443	0,1572	0,1575	0,1394
CReSA228-KX249755	0,1317	0,1369	0,1398	0,1405	0,1407	0,1222
CReSA261-KX249756	0,1090	0,1202	0,1705	0,1386	0,1371	0,1598
CReSA100-KX249753	0,1067	0,1133	0,1579	0,1586	0,1605	0,1615
B1-522-4w-OP688199	0,1377	0,1474	0,1669	0,1636	0,1672	0,1649
B3-785-9w-OP688221	0,1387	0,1483	0,1680	0,1619	0,1639	0,1615
Nu4a-OP822977	0,1034	0,1126	0,1714	0,1820	0,1839	0,1529
M3-OP822964	0,1739	0,1800	0,1780	0,1636	0,1622	0,1564
N5-OP822963	0,1445	0,1519	0,1530	0,1586	0,1589	0,1409
Nu1-OP822973	0,1711	0,1768	0,1729	0,1736	0,1756	0,1735
R1-OM893828	0,1896	0,1957	0,1860	0,1803	0,1856	0,1701
R2-OM893829	0,1897	0,1957	0,1859	0,1803	0,1856	0,1701
CReSA3267-JF276435	0,1313	0,1363	0,1383	0,1419	0,1421	0,1203
AUT13883-KT326148	0,1677	0,1730	0,1682	0,1553	0,1555	0,1478
Lena-JF802085	0,2047	0,2148	0,2097	0,2020	0,2007	0,1615
SU1Bel-KP889243	0,2089	0,2162	0,2092	0,1870	0,1940	0,1804
07V063-GU737264	0,1593	0,1673	0,1591	0,1603	0,1622	0,1357
13V091-KT159248	0,1704	0,1752	0,1686	0,1987	0,1973	0,1735
13V117-KT159249.1	0,1597	0,1678	0,1592	0,1603	0,1622	0,1357
BJEU061-GU047344.1	0,1556	0,1616	0,1535	0,1653	0,1672	0,1375
HeB3-MN927227	0,1567	0,1621	0,1531	0,1703	0,1689	0,1392
DK2011051114-KC862567	0,1389	0,1438	0,1353	0,1572	0,1608	0,1291
FR201456111-KY767026	0,1333	0,1393	0,1386	0,1452	0,1455	0,1289
FR201656111-MH018883	0,1367	0,1433	0,1423	0,1436	0,1455	0,1340
HU19401-MH463457	0,1648	0,1720	0,1678	0,1703	0,1689	0,1615
PR40/2014-MF346695	0,1813	0,1842	0,1778	0,1820	0,1823	0,1340
WestSib13-KX668221	0,2111	0,2204	0,2122	0,2154	0,2174	0,2285
CBNU0495-KY434183	0,1681	0,1728	0,1723	0,1753	0,1789	0,1564
***Porcilis PRRS*-KJ127878**	0,1326	0,1367	0,1281	0,1519	0,1522	0,1237
***PRRSFlex EU*-KT988004**	0,1572	0,1649	0,1548	0,1469	0,1472	0,1340
***Unistrain*-GU067771**	0,1315	0,1367	0,1391	0,1452	0,1455	0,1237
***Suvaxyn PRRS*-LQ787782**	0,1518	0,1560	0,1503	0,1753	0,1756	0,1237
***PYRSVAC183*-GQ461593**	0,1315	0,1369	0,1391	0,1469	0,1472	0,1271

Supplementary Table 7. Amino acid comparison of PRRSV-1 sequences variants 1α, 1β, and strain 2. The table shows the differences between the predicted amino acid composition of the different viral proteins inferred from the consensus nucleotide sequences of the variants 1α, 1β, and strain 2 (in rows). Columns indicate positions where amino acid differences were found in each examined protein. The amino acid position corresponds to the alignment with the prototype PRRSV-1 strain Lelystad (LV; NC_043487). Grey shaded cells show the position where a different amino acid was found between variants 1α and 1β. Yellow shaded cells indicate insertions or deletions.

nsp1α (n=18)

**Amino acid position**	24	36	42	55	58	71	79	84	95	112	113	114	127	138	140	142	162	169
**Strain 1α**	F	S	L	N	H	T	S	I	T	S	R	Q	D/N	V	V	S	L	C
**Strain 1β**	Y	·	P	·	Q	·	A	·	·	·	·	·	D	·	·	A	·	·
**Strain 2**	Y	P	T	D	H/R	S	·	L	L	A	S	R/K	D	M	L	A	S	R/C

nsp1β (n=40)

**Amino acid position**	8	9	13	16	17	18	21	26	28	30	33	34	37	43	46	50	60	62	67	69	72	77	78
**Strain 1α**	G	L	V	T	L	D	S	T	G	S	A	S	V	G	A	T	N	A	T	T	S	S	M
**Strain 1β**	·	·	·	·	·	·	·	·	·	·	·	·	·	·	·	·	·	·	·	·	·	·	·
**Strain 2**	N	S	M	L	R	N	T	A	E	N	V	N	A	I	V	I	D/N	S	V	S	H	G	T

**Amino acid position**	80	82	85	87	102	105	106	107	109	114	117	159	170	180	186	191	195
**Strain 1α**	Q	C	L	Q	F	P	A	E	W	H	G	P	E	M	S	V	V
**Strain 1β**	·	·	·	·	·	·	·	·	·	·	·	·	·	T	·	·	F
**Strain 2**	H	S	I/V	T	L	S	P	G	A	Y	N	S	G	M	P	T	V

nsp2 (n=209)

**Amino acid position**	8	9	12	14	15	16	18	19	20	21	25	26	28	31	33	34	40	48	53	58	66	69
**Strain 1α**	A	R	V	N	D	K	S	V	T	A	A	K	I	C	A	T	T	V	V	H	M	D
**Strain 1β**	·	·	·	·	·	·	·	·	·	·	·	·	·	·	·	·	·	·	·	·	·	N
**Strain 2**	T	K	A	S	K	G	S/L	T	A	S	V	P	I/T	Y	T	V	A	V/A	M	N	A	N

**Amino acid position**	72	78	82	83	88	98	107	108	122	132	133	143	147	148	149	151	152	160	170	174
**Strain 1α**	P	D	A	E	M	R	I	K	V	I	V	P	E	G	E	S	E	A	V	T
**Strain 1β**	·	·	·	·	·	·	·	R	·	·	·	·	·	·	·	·	·	·	·	·
**Strain 2**	E	D/C	T	Q	L	H	I/M	K	M	V	I	S	D	D	G	P	N	S	S	S

**Amino acid position**	178	182	184	187	200	203	230	231	239	248	249	262	268	270	272	274	275	276	278
**Strain 1α**	A	P	G	S	S	T	R	M	K	V	R	E	V	A	L	P	T	S	D
**Strain 1β**	·	·	·	·	S/P	·	·	·	·	·	·	·	·	·	·	·	·	·	·
**Strain 2**	N	Del	E	T	S	S	K	L	R/K/G	I	K	D	I/T	S	S	L	A	P	E

**Amino acid position**	280	282	284	285	289	288-298	300	301	302	303	305	306	308	309	311	313	315
**Strain 1α**	S	S	D	T	L		V	A	S	Q	A	Q	S	S	E	A	S
**Strain 1β**	·	·	·	·	L/S		·	·	·	·	·	·	·	·	·	·	·
**Strain 2**	P	N	G	A	A	Del	A	T	P	E	V	P	G	G	K	V	L

**Amino acid position**	317	319	320	321-358	362	363	364-490	406	420	436	437	491	492	497	501	504	509
**Strain 1α**	V	V	A	Del	G	G	Del	-	-	-	-	G	H	R/L	G	G/E	I
**Strain 1β**	·	·	·	·	·	·	·	-	-	-	-	·	·	·	·	·	·
**Strain 2**	P	T	K	-	S	E/G	-	G/S	P/S	G/E	S/L	S	D	L	E	E	T

**Amino acid position**	511	514	516	517	519	520	523	525	527	528	529	532	533	534	535	536	539
**Strain 1α**	L	M	D	A	V	A	S	G	T	T	P	V	S	S	G	A	L
**Strain 1β**	·	·	·	·	·	·	·	·	·	·	·	·	·	·	·	·	·
**Strain 2**	F	A	G	V	I	V	P	D	T/A	I	S	A	L	Q	E	V	F

Amino acid position	544	545	549	563	567	570	573	592	595	599	622	637	638	642	643	644	646
**Strain 1α**	L	K	Y	D	R	N	Y	D	N	E	K	R	K	N	D	C/Y	D
**Strain 1β**	·	·	·	·	·	·	·	·	·	·	·	·	·	·	·	·	·
**Strain 2**	P	N	F	G	K	S	F	A	D	N	D	K	K/R	S	S	N	V/I

Amino acid position	649	657	659	(660)	660	661	662	664	666	667	668	670	671	672	673	674	681
**Strain 1α**	Y	K	S		V	V	S	P	P	V	E	A	P	G	R	A	I
**Strain 1β**	·	·	·		·	·	·	·	·	·	·	·	·	·	·	·	·
**Strain 2**	Q	K/R	G	Ins G	P	L/P	P	Q	S	T	G	V	F	N	Q	T	T

Amino acid position	682	683	684	685	686	687	688	689	691	694	695	697	698	699	702	703	704
**Strain 1α**	P	L	E	G	V	T	T	P	G	R	A	G	L	P	V	D	T
**Strain 1β**	·	·	·	·	·	·	·	·	·	·	·	·	·	·	·	·	·
**Strain 2**	R	Q	G	N	T	A	P	L	M	H	V	D	P	G	A	G	E

Amino acid position	705	706	710	711	712	713	716	720	721	722	723	726	727	729	734	741	750
**Strain 1α**	G	G	D	R	M	L	A	E	L	V	G	L	T	R	F	M	V
**Strain 1β**	·	·	·	·	·	·	·	·	·	·	·	·	·	·	·	·	A
**Strain 2**	G/E	G/R	N	L	V	I	T	G	S	I	S	S	M	W	V	A	S

Amino acid position	753	761	765	768	770	771	774	788	812	824	836	843	852	856	858	865	869
**Strain 1α**	S	V	V	F	R	L	A	V	D	K	Q	G	S	F	R	H	I
**Strain 1β**	·	·	·	·	·	·	·	·	·	·	·	·	·	·	·	·	·
**Strain 2**	P	I	A	L	Y/H	S	I	L	N	E	E	T	L	L	K	Y	V

Amino acid position	871	881	883	884	920	931	945	949	957	988	1003	1005
**Strain 1α**	L	L	I	I	A	S	H	C	S	I	L	T
**Strain 1β**	·	·	·	·	·	·	·	·	·	·	·	·
**Strain 2**	F	F/L	L	V	T	A	R	R	P	V	Q	V

nsp3 (n=34)

Amino acid position	12	18	21	39	54	58	59	60	63	64	65	75	76	91	102	107	117	123	127	137	139
**Strain 1α**	N	N	V	T	I	S	S	T	R	A	T	A	Q	F	P	S	P	P	A	Y	A
**Strain 1β**	·	·	·	·	·	·	·	·	·	·	·	·	·	·	·	·	·	·	·	·	·
**Strain 2**	K	E	I	A	T	N	P	A	K	T	V	V	H	L	A	A/T	P/S	V	F	T	I

Amino acid position	152	156	159	160	172	179	180	185	215	223	234	235	292
**Strain 1α**	V	I	T	A	F	G	G	M	V	V	V	T	I
**Strain 1β**	·	·	·	·	·	·	·	·	·	·	·	·	·
**Strain 2**	I	V	V	T	L	C	A	A/V	M	I	A	V	V

nsp4 (n=29)

Amino acid position	6	10	31	33	34	38	45	52	55	58	60	69	70	72	74	76	79	81	82	83	85	105
**Strain 1α**	R	P	K	I	V	T	A	S	R	T	R	D	A	N	Q	V	A	K	V	V	G	E
**Strain 1β**	·	·	·	·	·	·	·	·	·	·	·	·	·	N/D	·	·	·	·	·	·	·	D
**Strain 2**	H	L	R	T	I	A	T	E	C	T/V	K	H	V	N/D	P	I	V	N	I	A	R	N

Amino acid position	113	129	147	178	180	188	201
**Strain 1α**	S	I	D	G	T	I	V
**Strain 1β**	·	·	·	·	·	·	·
**Strain 2**	N	V	E	S	A	V	T/I

nsp5 (n=14)

Amino acid position	26	37	57	86	89	98	102	105	120	122	148	151	152	158
**Strain 1α**	V	I	L	L	I	T	R	D	I	M	H	A	M	A
**Strain 1β**	·	·	·	·	·	·	·	·	·	·	·	·	·	·
**Strain 2**	I	V	V	F	V	V	K	E	L	V	C	D	T	H

nsp6 (n=1)

Amino acid position	5
**Strain 1α**	R
**Strain 1β**	·
**Strain 2**	K

nsp7a (n=10)

Amino acid position	20	51	65	67	79	106	116	134	138	139
**Strain 1α**	S	R	V	S	S	R	N	N	D	V
**Strain 1β**	·	·	·	·	P	·	·	·	·	·
**Strain 2**	N	Q	I	A	S	K	S	D	G	I

nsp7b (n=17)

Amino acid position	7	26	28	37	48	53	57	64	65	69	76	77	78	88	90	94	110
**Strain 1α**	C	F/L	S	V	T	Y	Y	A/V	N	R	V	T	S	T	A	I	I
**Strain 1β**	·	F	N	·	·	·	·	A	·	·	·	·	·	·	·	·	·
**Strain 2**	S	F	N	I	N	Y/F	H	A	D	K	I	D	H	I	V	V	V

nsp8 (n=3)

Amino acid position	3	32	39
**Strain 1α**	R	D	T
**Strain 1β**	·	·	·
**Strain 2**	K	N/D	T/N

nsp9 (n=31)

Amino acid position	1	2	29	49	51	55	66	106	109	119	148	157	158	162	169	178	188	192	221	232
**Strain 1α**	T	S	V	V	V	T	S	D	I	V	Y	H	A	T	L	G	H	A	M	P
**Strain 1β**	·	·	·	·	·	·	·	·	·	A	·	·	·	·	·	·	·	·	·	·
**Strain 2**	A	G	I	A	I	I	C	D/N	V	A/V	H	Y	Q	I	I	R/K	Q	V/A	V	F/L

Amino acid position	234	241	270	311	339	413	465	487	543	557	584
**Strain 1α**	F	R	V	I	L	A	I	G	T	R	A
**Strain 1β**	·	K/R	·	·	·	·	·	G/S	·	·	·
**Strain 2**	C	K	I	V	P	V	V	G	K	K	T

nsp10 (n=32)

Amino acid position	2	9	16	40	47	51	59	61	64	66	84	89	116	180	182	216	217	261	266	325	326
**Strain 1α**	R	V	H	N	A	S	A	K	I	N	C	V	P	V/I	S	Y	T	Y	D	I	G
**Strain 1β**	·	·	·	·	·	·	·	·	·	·	·	·	·	V	·	·	·	·	·	·	·
**Strain 2**	K	I	Y	S	S	P	T	R/K	L	A	S	A	A	I	C	H	V	C	N	T	G/D

Amino acid position	341	354	375	378	382	387	404	406	420	428	429
**Strain 1α**	I	K	N	Q	D	R	N	D	S	L	R
**Strain 1β**	·	·	·	K	·	·	·	·	·	P	·
**Strain 2**	V	R	D	Q	N	H	D	D/N	P	P	K

nsp11 (n=10)

Amino acid position	23	40	59	71	76	92	98	119	120	155
**Strain 1α**	A	H	G	V	T	I	A	T	A	F
**Strain 1β**	·	·	·	·	·	·	·	·	·	·
**Strain 2**	V	S	A	A	I	V/I	G	A	T	T

nsp12 (n=20)

Amino acid position	2	42	57	59	81	83	84	88	97	100	106	115	118	138	145	146	147	148	149	151
**Strain 1α**	L	D	Q	V	R	S	L	S	R	N	I	N	F	Q	Q	L	P	P/S	E	E
**Strain 1β**	·	·	·	·	·	·	·	·	·	·	·	·	·	R	·	·	·	·	·	·
**Strain 2**	F	N	K	I	K/R	T	F	N	C	D	T	E	C	Q	R	S	L	L	G	A

GP2 (n=35)

Amino acid position	8	11	13	16	22	24	25	26	27	29	30	32	36	41	42	67	80	83	88	98	102	106
**Strain 1α**	V	A	C	M	S	A	W	L	T	S	F	L	L	P	D	S	R	A	F	E	H	E
**Strain 1β**	·	·	·	·	·	·	·	·	·	·	·	·	S	·	·	·	K	·	·	·	R	D
**Strain 2**	A	P	Y	T	L	V	W/L	S	I	L	F/S	W	L	Q	V	N	R	V	I	M	H	E

Amino acid position	135	138	141	170	177	181	187	192	194	232	235	244	248
**Strain 1α**	K	R	I	V	L	T	L	S	S	A	Y	V	S
**Strain 1β**	·	·	·	·	·	·	·	·	·	·	·	·	·
**Strain 2**	R	N	V	A	Q	S	I/L	P	T	V	C	A	L

E (n=3)

Amino acid position	9	61	69
**Strain 1α**	S	V	I
**Strain 1β**	·	·	·
**Strain 2**	T	I	V

GP3 (n=58)

Amino acid position	3	4	7	10	11	12	14	15	18	23	30	46	48	60	64	76	80	81	91	92	93	100
**Strain 1α**	Y	Q	R	F	L	L	S	F	Y	A	T	Q	A	R	M	R	D	H	L	M	P	D
**Strain 1β**	·	·	·	·	·	·	·	·	·	·	·	·	T	·	·	·	·	·	·	·	·	N
**Strain 2**	H	K	C	F/L	F	F	G	L	H	T	F/S/T	E	T	S	Y/H	K	T	T	S	L	S	N

Amino acid position	102	127	139	140	142	146	154	156	157	158	166	172	204	207	211	215	216	217	218
**Strain 1α**	R	G	G	R	A	E	V	A	N	H	V	I	A	A	I	F	K	P	I
**Strain 1β**	·	·	·	·	·	·	·	·	·	·	·	·	·	·	·	·	·	·	·
**Strain 2**	K	G/R	R	H	F	V	I	T	T	Y	A	V	V	V	T	L	R	L	T

Amino acid position	224	232	234	235	236	237	239	244	245	247	248	250	252	261	262	263	265
**Strain 1α**	V	S	S	D	I	Del	S	Del	K	L	L	N	H	L	N	T	R
**Strain 1β**	·	·	P	·	·	·	·	·	·	·	·	·	·	·	·	·	·
**Strain 2**	G	I	S	N	H	T	P	G	A	P	Del	Y	P	P	S	I	Q

GP4 (n=28)

Amino acid position	8	11	12	14	29	35	50	51	53	54	55	57	58	63	64	65	66	67	68	69	71	128
**Strain 1α**	F	S	T	Y	P	Q	I	E	L	R	Del	W	V	E	G	Del	A	I	R	K	S	T
**Strain 1β**	·	·	·	·	·	·	·	·	P	·	·	·	·	·	·	·	·	·	·	·	·	·
**Strain 2**	L	G	A	H	T	K	L	D	·	Q	P/L	R	A	R	S	S	P	Del	G	V	A	V

Amino acid position	143	149	150	154	162	179
**Strain 1α**	I	Y	H	D	L	I
**Strain 1β**	·	·	·	·	·	·
**Strain 2**	T	H	Y	N	M	V/I

GP5 (n=40)

Amino acid position	2	4	6	7	9	11	13	14	17	18	20	22	25	31	33	35	46	56	58	59	60	79	93
**Strain 1α**	R	L	K	L	L	S	L	H	F	W	F	F	I	S	D	S	N/D	N	L	F	G	I	L
**Strain 1β**	·	·	·	·	·	·	·	·	P	·	·	·	·	·	·	·	N	·	Del	·	·	·	·
**Strain 2**	K	S	R	S	H	L	P	Y	C	C	L	L	T	F	V	N	N	A	L	R/H	K	L	F

Amino acid position	96	97	101	104	105	106	115	122	123	125	130	162	166	173	174	182	193
**Strain 1α**	G	A	I	Y	D/G	G	C	L	T	L	V	V	S	S	N	V	R
**Strain 1β**	·	·	·	·	G	·	·	·	·	·	·	·	·	·	·	·	·
**Strain 2**	S	V	A	H	D	G/R	G	F	V	F	A	I	L	G	D	I	K

M (n=20)

Amino acid position	3	6	9	11	14	18	28	62	65	74	80	84	87	94	100	123	130	152	161	165
**Strain 1α**	S	N	G	P	A	V	I	A	E	L	F	V	F	V	M	P	R	S	V	L
**Strain 1β**	·	·	·	·	·	·	·	·	·	·	·	·	·	·	·	·	·	·	·	·
**Strain 2**	G	N/D	Y	S	V	A	V	E	Q	M	L	I	L	I	L	S	Q	G	V/I	V

N (n=17)

Amino acid position	7	10	12	20	23	33	34	35	40	41	70	90	100	122	126	127	128
**Strain 1α**	G	N	Q	S	I	V	M	R	R	P	L	A	S	S	N	V	D
**Strain 1β**	·	·	·	·	·	·	·	·	·	·	·	·	·	·	·	·	·
**Strain 2**	S	K	K	G	V	M	I	K	Q	S	Q	V	G	S/P	G	A	N

Supplementary Figure 1. Bayesian tree showing the phylogenetic grouping of the ORF5 sequences obtained in this study. Posterior probabilities higher than 70% are shown. All isolates from batches 0 and 1 belonged to the 1α cluster (red). All isolates of batches 2 to 4 and most of the Batch 5 sequences belonged to variant 1β (green). In Batch 5, a new strain appeared in the nurseries and subsequently became the only detectable PRRSV strain in batches 6 and 7 (strain 2, in blue).

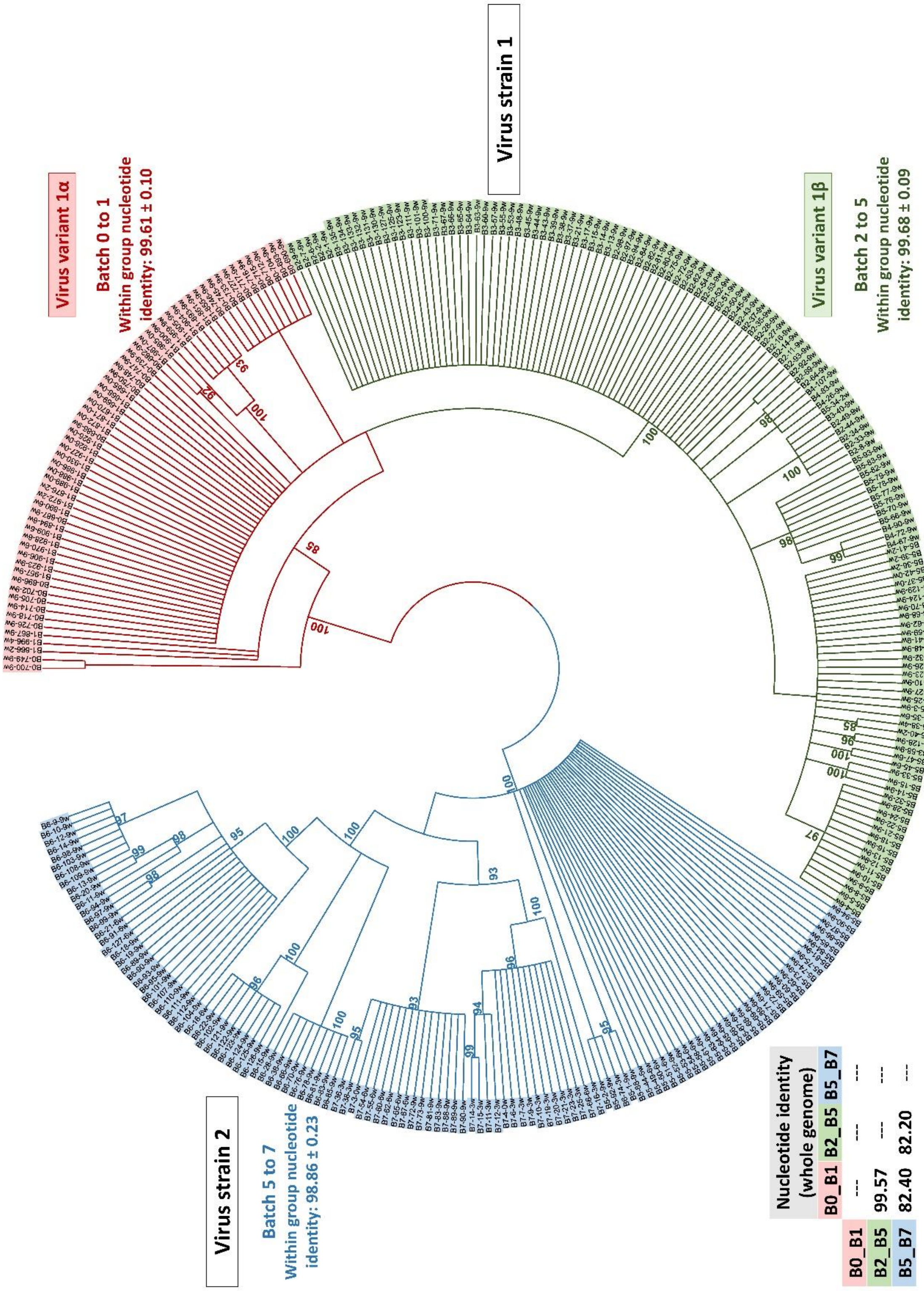

Supplementary Figure 2. Bayesian phylogenetic tree constructed using the whole genome sequences of PRRSV-1. The red and green shaded areas denote the sequences that were obtained from Strain 1 and 2, respectively. Lelystad strain is the prototype for PRRSV-1. The strains that are coloured in red represent the commercially licensed vaccines in Spain. Only posterior probability values >70% are shown.

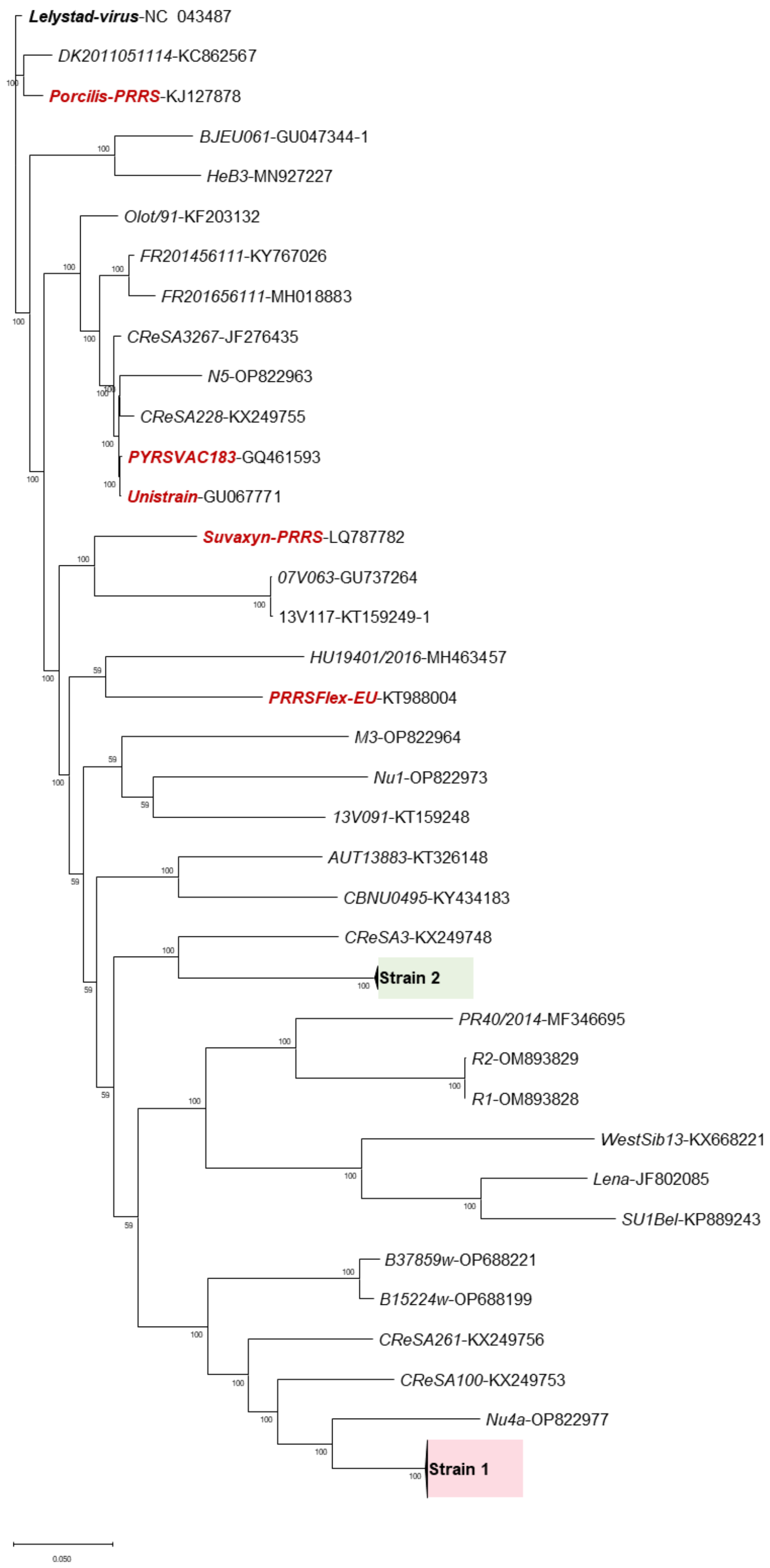

Supplementary Figure 3. Bayesian phylogenetic tree based on ORF5 sequences of PRRSV-1. The red and green shaded areas represent sequences from Strain 1 and 2, respectively. Lelystad strain is the prototype for PRRSV-1. The red-coloured strains indicate vaccines licensed in Spain. Only posterior probability values >70% are shown.

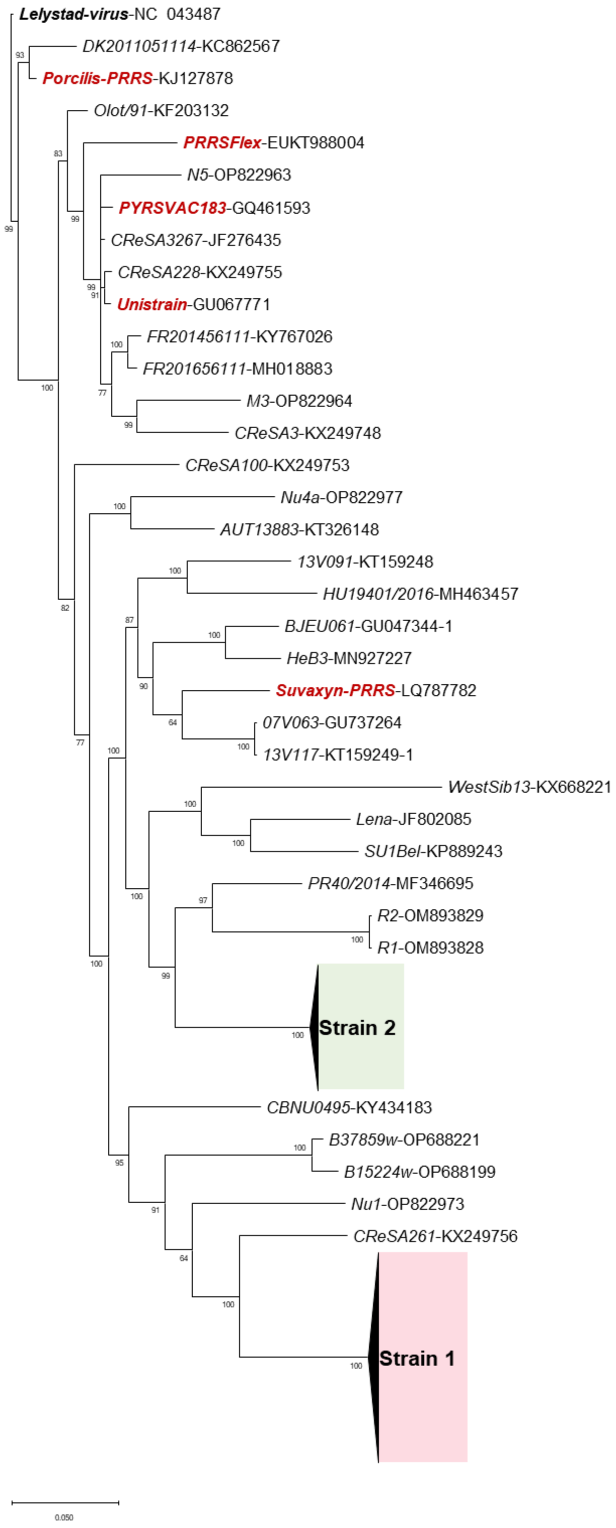

Supplementary Figure 4. Genome recombination event between variants 1α and 1β. Phylogenetic trees constructed by GARD based on the resulting fragments from the recombination event in nsp2 (nucleotide positions 2,335-2,958). The Akaike Information Criterion (AIC) scores for the non-recombinant and the recombinant trees were 1,086.2 and 904.0, respectively. Isolates of variant 1α are shown in red and of variant 1β in green. Nucleotide position correspond to the alignment with the prototype PRRSV-1 strain Lelystad (LV; NC_043487).

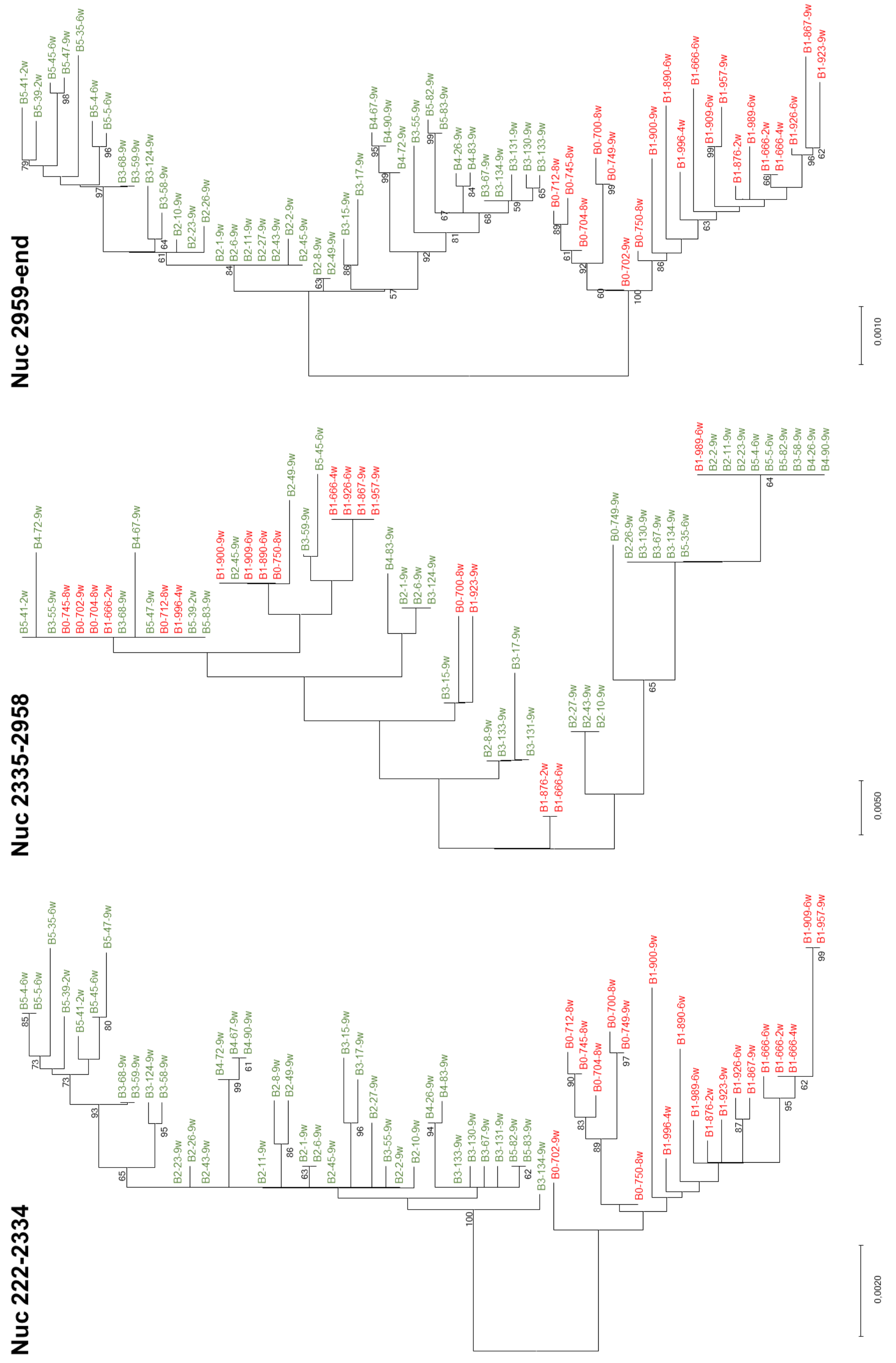

Supplementary Figure 5. Example of the genetic distance plots for a recombination event. The plots illustrate the signals detected in ORF1a for one of the recombinant isolates (B2-52-9w). A) Distance plot between parental strain 1α and parental strain 1β; B) Distance plot between recombinant and parental strain 1β; C) Distance plot between recombinant and parental strain 1α.

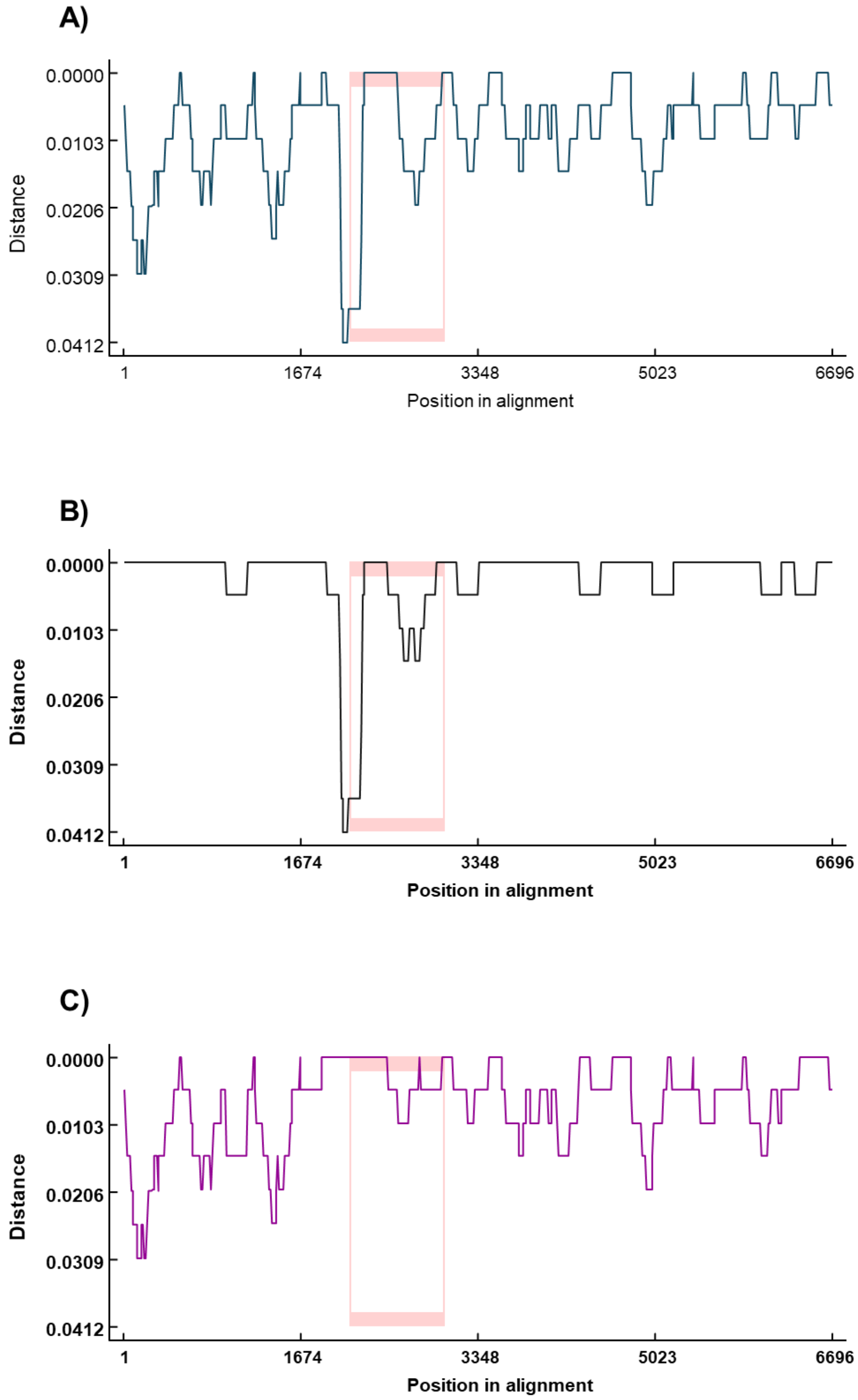

Supplementary Figure 6. Antibody levels of six- and nine-week-old piglets against the viral nucleocapsid protein expressed as S/P ratios as determined in ELISA. Each dot represents an individual. A) Animals examined at six weeks of age; B) Animals examined at nine weeks of age. ns=non-significant differences. *p<0.05; **p<0.01; ***p<0.001; ****p<0.0001.

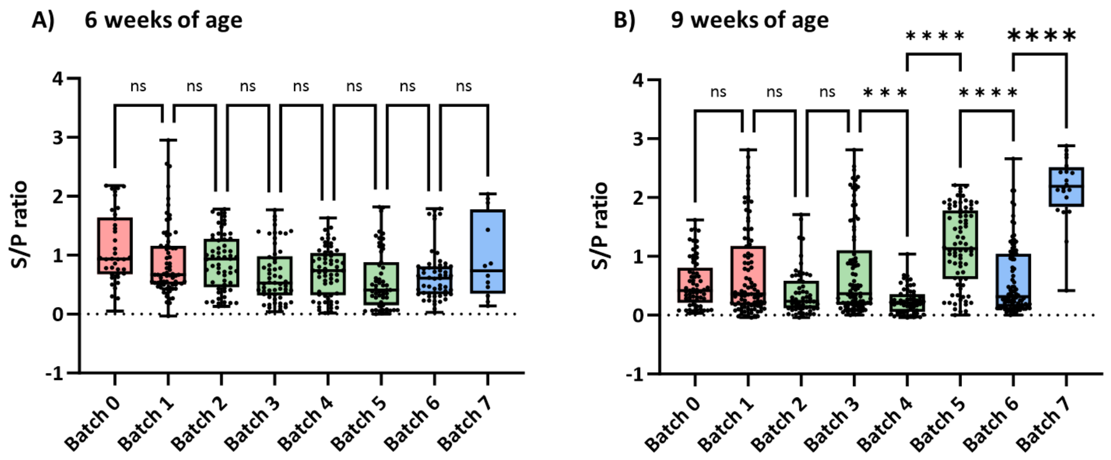

Supplementary Figure 7. Neutralizing antibody titres in sows from Batch 1 against the variants 1α and 1β and the vaccine virus. Comparison of neutralization titres (log_2) obtained for each sow from Batch 1 with variants 1α (A) or 1β (B) with the titres against the vaccine used in the farm. The diagonal (dotted red line) represents the line of identity for both tests.

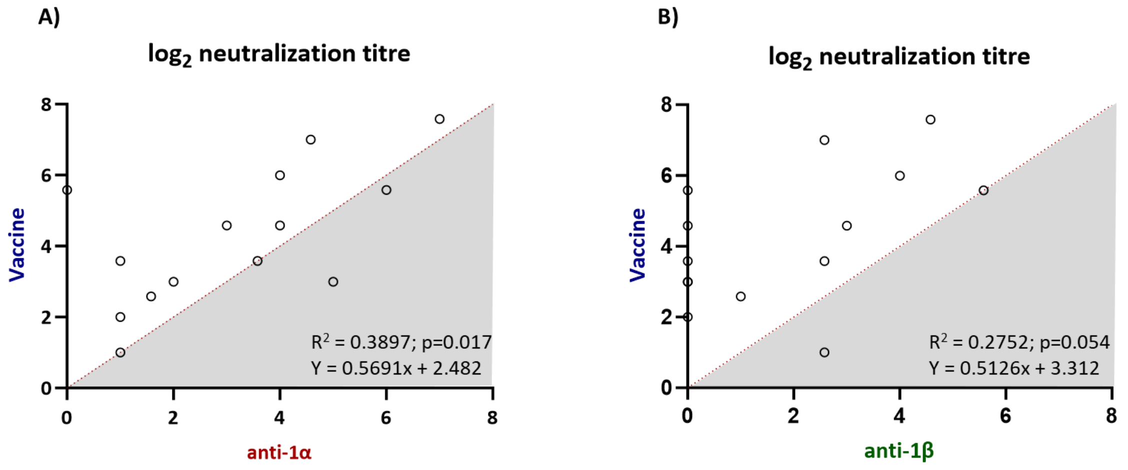

Supplementary Figure 8. Inhibition of the levels of poly I:C induced IFN-α and TNF-α in PAM. The graphs show the levels of IFN-α (A) and TNF-α (B) in the cell supernatants of PAM after being inoculated with the variants 1α and 1β in different treatment combinations with poly I.C. virus+Poly I:C= both simultaneously; virus+Poly I:C 6h= virus followed by oly I:C 6h later; Poly I:C+virus 6h= poly I:C followed by virus 6h later. *p<0.05; **p<0.01; ***p<0.001; ****p<0.0001.

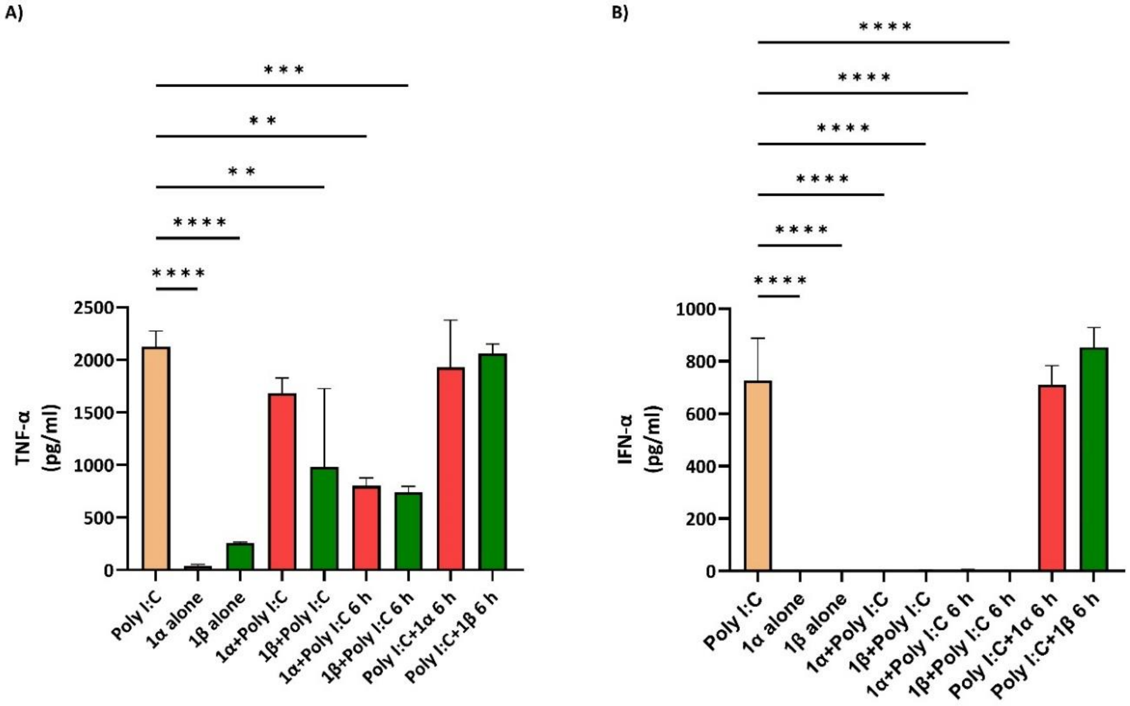

Supplement: veae041_Supp [file veae041_supp.zip › suppl_data/Supplementary_material_final.pdf]
